# Fast and efficient copper-mediated ^18^F-fluorination of arylstannanes, aryl boronic acids, and aryl boronic esters without azeotropic drying

**DOI:** 10.1186/s41181-019-0079-y

**Published:** 2019-10-16

**Authors:** Salla Orvokki Lahdenpohja, Noora Annika Rajala, Johan Rajander, Anna Kaarina Kirjavainen

**Affiliations:** 1 Radiopharmaceutical Chemistry Laboratory, Turku PET Centre, University of Turku, Kiinamyllynkatu 4-8, 20520 Turku, Finland; 2 Accelerator Laboratory, Turku PET Centre, Åbo Akademi University, Porthaninkatu 3, 20500 Turku, Finland

**Keywords:** Radiolabelling, Fluorine-18, Aryl stannanes, Aryl boronic acids, Copper-mediated, Azeotropic drying-free

## Abstract

**Background:**

Copper-mediated radiofluorination is a straightforward method to produce a variety of [^18^F]fluoroarenes and [^18^F]fluoroheteroarenes. To minimize the number of steps in the production of ^18^F-labelled radiopharmaceuticals, we have developed a short and efficient azeotropic drying-free ^18^F-labelling method using copper-mediated fluorination. Our goal was to improve the copper-mediated method to achieve wide substrate scope with good radiochemical yields with short synthesis time.

**Results:**

Solid phase extraction with Cu (OTf)_2_ in dimethylacetamide is a suitable activation method for [^18^F]fluoride. Elution efficiency with Cu (OTf)_2_ is up to 79% and radiochemical yield (RCY) of a variety of model molecules in the crude reaction mixture has reached over 90%. Clinically relevant molecules, norepinephrine transporter tracer [^18^F]NS12137 and monoamine transporter tracer [^18^F]CFT were produced with 16.5% RCY in 98 min and 5.3% RCY in 64 min, respectively.

**Conclusions:**

Cu (OTf)_2_ is a suitable elution agent for releasing [^18^F]fluoride from an anion exchange cartridge. The method is fast and efficient and the Cu-complex is customizable after the release of [^18^F]fluoride. Alterations in the [^18^F]fluoride elution techniques did not have a negative effect on the subsequent labelling reactions. We anticipate this improved [^18^F]fluoride elution technique to supplant the traditional azeotropic drying of [^18^F]fluoride in the long run and to concurrently enable the variations of the copper-complex.

## Background

Positron emission tomography (PET) and related hybrid methods (PET/MRI and PET/CT) are essential tools for in vivo imaging. Among radionuclides used for PET, fluorine-18, a short-lived radioisotope of fluorine, has proven to be advantageous owing to the adequate half-life (109.8 min), clean decay process (97% β^+^), and low positron energy (634 keV). These properties tolerate multistep synthesis and purification. Introduction of fluorine-18 to the suitable molecular probes has remained a challenge owing to the lack of suitable ^18^F-labelling methods (Miller et al. 2008).

Traditionally, ^18^F-fluorination reactions have been divided in to two categories, electrophilic and nucleophilic radiofluorination. The electrophilic fluorination reagent, [^18^F]F_2_, is advantageous when labelling electron-rich arenes, but the produced radiotracers are always achieved with low molar activity (A_m_). Low A_m_ is due to the use of added carrier-fluorine in the production of [^18^F]F_2_. Thus, nucleophilic ^18^F-fluorination with [^18^F]fluoride has been the most widely used radiofluorination method all over the world. However, the reactivity scope of the nucleophilic substitution has been confined to electron-poor arenes (Preshlock et al. 2016). The reactivity scope of the nucleophilic radiofluorination has been improved by the use of iodonium salts and ylides (Pike and Aigbirhio 1995; Ross et al. 2007; Satyamurthy and Barrio 2010) and sulfonium salts (Mu et al. 2012; Gendron et al. 2018). Recently, transition metal mediated radiofluorination with palladium (Lee et al. 2011; Kamlet et al. 2013) and nickel (Lee et al. 2012; Zlatopolskiy et al. 2015b; Hoover et al. 2016) has been introduced. Palladium-mediated fluorination has proved to be impractical due to the moisture sensitive nature of the required palladium complexes. Additionally, both of these methods involve the use of complex precursor molecules, which have proven to be challenging to synthesize. Another challenge includes the automation of the synthetic procedures of nickel- and palladium-mediated ^18^F-labelling. Automation of the synthesis procedures is essential for production of radiopharmaceuticals according to good manufacturing practice (GMP).

A most promising development of the radiofluorination methods was the introduction of the copper-mediated ^18^F-fluorination. Via this method, a wide scope of ^18^F-labelled arenes and heteroarenes has been synthesized starting from simple arylboronic esters (Tredwell et al. 2014), aryl boronic acids (Mossine et al. 2015), or stannylated arenes (Makaravage et al. 2016) with straightforward synthesis conditions. Copper-mediated ^18^F-fluorination has been widely studied in many radiochemistry laboratories around the world and it has been optimized to achieve high radiochemical yields (RCY) and good A_m_ values by improving the reaction conditions. Copper-mediated fluorination typically involves the use of a base. For example, when [^18^F]fluoride is dried traditionally with conventional azeotropic drying, K_2_CO_3_ (pKa of the conjugate acid is 10.3) and cryptand, Kryprofix_222_, are used. Additionally, the copper-complex usually used for the radiolabelling reaction contains pyridine (Cu(OTf)_2_(py)_4_, with the pKa of the conjugate acid of pyridine being 5.3). Use of bases and/or cryptands have been blamed for suppression of the RCYs (Zlatopolskiy et al. 2015a; Antuganov et al. 2017; Mossine et al. 2017). In addition, subsequent reactions might be sensitive to bases. To avoid the use of these reagents, alternative [^18^F]fluoride processing methods have been reported (Mossine et al. 2017; Zischler et al. 2017). Furthermore, the use of azeotropic drying is a time consuming process and results in part of the [^18^F]fluoride adhering in the glass vessel walls during the drying process (Mossine et al. 2015). Consequently, the latest developments include the replacement of azeotropic drying of [^18^F]fluoride by solid phase extraction (SPE). The SPE process has been improved by using alcohols in the [^18^F]fluoride processing (Zischler et al. 2017) or pyridinium sulfonates (Antuganov et al. 2019) or dimethylaminopyridinium triflates (DMAP) as an elution agents (Zhang et al. 2019). However, these new [^18^F]fluoride elution methods typically involve separate evaporation steps after the elution or utilize relatively toxic chemicals, like DMAP, which might not be necessary for the radiolabelling reaction itself. In the clinical radiopharmaceutical production, it is preferred to minimize the use of different, possibly toxic reagents or chemicals to keep the final purification of the radiotracer as simple as possible.

Recently, our group improved the copper-mediated radiofluorination conditions to quickly and efficiently produce norepinephrine transporter tracer [^18^F]NS12137 (*exo*-3-[(6-[^18^F]fluoro-2-pyridyl)oxy]8-azabicyclo [3.2.1]octane) (Lahdenpohja et al. 2019). Herein, we introduce an azeotropic drying-free copper-mediated radiofluorination method, where the copper-complex is customizable after the drying of [^18^F]fluoride by SPE and the [^18^F]fluoride is ready to use in subsequent labelling reaction without the use of additional reagents or any other manipulations. Our aim was to study the effects of the preconditioning of different typically used SPE cartridges on trapping and elution of [^18^F]fluoride. We used Cu(OTf)_2_ and Cu(OTf)_2_(py)_4_ for the elution of [^18^F]fluoride, and labelled several arenes and heteroarenes, including [^18^F]NS12137 and monoamine transporter tracer [^18^F]CFT, to prove the usefulness of this method.

## Methods

### General

Unless otherwise stated in the supporting information, all of the reagents and solvents were used as received from commercial suppliers without further purification. The following, widely used in clinical radiotracer production, anion exchange cartridges with luers were used: Sep-Pak Accell Plus QMA Carbonate Plus Light Cartridge 46 mg; Sep-Pak Accell Plus QMA Plus Light Cartridge 130 mg and Chromafix PS-HCO_3_ 45 mg. Additionally we used handmade 10 mg QMA cartridges to see the effect of minimized anion exchange resin mass. The columns were preconditioned with following standard preconditioning solutions: 1) 20 mL H_2_O; 2) 10 mL 0.5 M LiOTf and 20 mL H_2_O; or 3) 10 mL 0.5 M Na_2_SO_4_ and 20 mL H_2_O. HPLC methods are described in the Supporting information Additional file 1.

### Radiochemistry studies

The full description of the elution and radiolabelling studies can be found in the supporting information. [^18^F]Fluoride elution and the radiolabelling reactions were performed in two hot cells with a remote-controlled synthesis devices that were built in-house. The process was not fully automated and the synthesis times of [^18^F]NS12137 and [^18^F]CFT are not optimised.

[^18^F]Fluoride was loaded to an anion exchange cartridge, the cartridge was washed with dimethylacetamide (DMA, 5 mL) and [^18^F]fluoride was eluted with Cu(OTf)_2_ or Cu(OTf)_2_(py)_4_ in DMA (0.5 mL). The amount of the copper-complex varied between 12 and 96 μmol to see the effect of the amount of the elution agent to the [^18^F]fluoride elution. In the preliminary test, LiOTf (24 μmol) was added to the elution solution. After elution with copper-complex, the cartridge was washed with DMA (0.5 mL). The elution efficiency (EE) was calculated by dividing the activity of the eluted [^18^F]fluoride fraction by the sum of the activity of the eluted [^18^F]fluoride fraction and the activity remaining in the SPE cartridge. The [^18^F]fluoride recovery was calculated by dividing the activity of the eluted [^18^F]fluoride fraction by the sum of the activity of the SPE cartridge and the waste bottle after loading the anion exchange cartridge. The EE and the [^18^F]fluoride recovery were non-decay-corrected because the [^18^F]fluoride loading, elution and the radioactivity measurements were completed within 5 min.

In the radiolabelling test, [^18^F]fluoride was eluted straight into a conical vial containing the precursor (8 μmol) and pyridine (50 μL) in DMA (50 μL), the total volume being 1.1 mL. The reaction solution was heated at 120 °C for 5 to 15 min under ambient air. For [^18^F]NS12137, a previously published deprotection method was followed (Kirjavainen et al. 2018). [^18^F]NS12137 and [^18^F]CFT were purified with semi-preparative HPLC.

[^18^F]Fluoride incorporation in [^18^F]NS12137 synthesis was determined according to SPE. The radioactivity in the reaction vial before loading the activity to the cartridge was compared to the activity eluted from the cartridge. All reported RCY values were decay-corrected to the end of bombardment (EOB). The RCY (based on radio-HPLC analysis of the crude reaction mixture) values were determined from the amount of overall radioactivity eluted from the analytical HPLC column. We verified that there was no leftover radioactivity in the injector or in the HPLC column after the analytical HPLC run. RCY (based on radio-HPLC analysis of the crude reaction mixture) values are expressed as mean ± standard deviation.

Levels of copper in [^18^F]NS12137 and [^18^F]CFT were analysed with inductively coupled plasma mass spectrometry (ICP-MS, PerkinElmer, Elan DRC Plus). Commercial multielement standard was used for instrument calibration.

## Results

### [^18^F]fluoride processing

[^18^F]Fluoride can be satisfactorily eluted from various SPE cartridges by using solely Cu(OTf)_2_(py)_4_ or Cu(OTf)_2_ as an elution agent in DMA. The term elution efficiency is used when the EE and the [^18^F]fluoride recovery are on the same level, which means no [^18^F]fluoride has been lost during trapping or washing processes. The term [^18^F]fluoride recovery is used when some of the [^18^F]fluoride is lost during the trapping and/or washing process. Preliminary [^18^F]fluoride elution tests showed that preconditioning of the SPE cartridge had, in some cases, a significant effect on the [^18^F]fluoride recovery when using copper complexes as eluting agents (see Table 1). Preconditioning of non-carbonated 130 mg QMA cartridges and carbonated 46 mg QMA cartridges with aqueous LiOTf solution resulted in higher [^18^F]fluoride recovery and the EE (Table 1, entries 5, 9) than with only aqueous preconditioning (Table 1, entries 7, 11). At the same time, we noticed, that using LiOTf and a copper complex together as an elution agent improved the [^18^F]fluoride elution when non-carbonated 130 mg QMA cartridges and carbonated 46 mg QMA cartridges were preconditioned only with water (Table 1, entries 8, 12). If these cartridges were preconditioned with aqueous LiOTf, additional LiOTf as an elution agent did not have a noteworthy effect on the EE (Table 1, entries 6, 10). With non-carbonated 10 mg QMA cartridges, the effect of the LiOTf preconditioning or use of LiOTf as an elution agent was unnoticeable (Table 1, entries 1–4). LiOTf preconditioning did not improve the [^18^F]fluoride elution in bicarbonated PS-HCO_3_ cartridges (Table 1, entries 15, 16). When using aqueous Na_2_SO_4_ solution for the preconditioning of a carbonated cartridge, [^18^F]fluoride recovery was only 0.6 ± 0.1% and the EE 3.5 ± 0.5% (*n* = 2) (Table 1, entry 14). The elution speed did not have a significant effect on the EE (Table 1, entries 11, 13). The only case when a significant difference was observed between [^18^F]fluoride recovery and EE was when 10 mg QMA cartridges were used (Table 1, entries 1–4).

**Table 1:** Preliminary results of [^18^F]fluoride elution studies with various preconditioning and elution agents

Entry (*n* ≥ 2)	Cartridge	Cartridge preconditioning	Elution agent^a^	Elution speed	Fluoride recovery ± SD (%)	EE^b^ ± SD (%)
1	QMA 10 mg	0.5 M LiOTf, H_2_O	Cu(OTf)_2_(py)_4_	slow	57.8 ± 3.4	71.0 ± 4.4
2	QMA 10 mg	0.5 M LiOTf, H_2_O	Cu(OTf)_2_(py)_4_, LiOTf (1:1)	slow	60.2 ± 5.9	73.5 ± 3.5
3	QMA 10 mg	H_2_O	Cu(OTf)_2_(py)_4_	slow	55.8 ± 0.6	65.7 ± 0.4
4	QMA 10 mg	H_2_O	Cu(OTf)_2_(py)_4_, LiOTf (1:1)	slow	57.4 ± 2.2	68.6 ± 1.9
5	QMA 130 mg	0.5 M LiOTf, H_2_O	Cu(OTf)_2_(py)_4_	slow	47.1 ± 9.7	52.1 ± 11.3
6	QMA 130 mg	0.5 M LiOTf, H_2_O	Cu(OTf)_2_(py)_4_, LiOTf (1:1)	slow	60.3 ± 2.7	67.3 ± 1.2
7	QMA 130 mg	H_2_O	Cu(OTf)_2_(py)_4_	slow	40.1 ± 0.0	45.6 ± 0.6
8	QMA 130 mg	H_2_O	Cu(OTf)_2_(py)_4_, LiOTf (1:1)	slow	63.8 ± 4.2	73.6 ± 3.0
9	QMA carb 46 mg	0.5 M LiOTf, H_2_O	Cu(OTf)_2_(py)_4_	slow	41.8 ± 5.2	44.6 ± 5.6
10	QMA carb 46 mg	0.5 M LiOTf, H_2_O	Cu(OTf)_2_(py)_4,_ LiOTf (1:1)	slow	42.7 ± 4.4	46.1 ± 4.3
11	QMA carb 46 mg	H_2_O	Cu(OTf)_2_(py)_4_	slow	25.3 ± 5.3	25.1 ± 3.0
12	QMA carb 46 mg	H_2_O	Cu(OTf)_2_(py)_4,_ LiOTf (1:1)	slow	38.6 ± 2.1	42.7 ± 3.8
13	QMA carb 46 mg	H_2_O	Cu(OTf)_2_(py)_4_	fast	19.9 ± 0.6	20.7 ± 0.8
14	QMA carb 46 mg	0.5 M Na_2_SO_4_, H_2_O	Cu(OTf)_2_(py)_4_	slow	0.6 ± 0.1	3.5 ± 0.4
15	PS-HCO_3_ 45 mg	0.5 M LiOTf, H_2_O	Cu(OTf)_2_(py)_4_	slow	24.8 ± 14.6	37.2 ± 10.9
16	PS-HCO_3_ 45 mg	H_2_O	Cu(OTf)_2_(py)_4_	slow	31.6 ± 9.1	35.3 ± 10.6

^a^24 μmol per elution agent

^b^EE = elution efficiency

The results from the [^18^F]fluoride elution studies with various cartridges, cartridge preconditioning, and amounts of the elution agent are presented in Table 2. Entries with the lowest and the highest elution efficiencies are highlighted. The EE of [^18^F]fluoride varied from 18.5% (Table 2, entry 7) to 79.5% (Table 2, entry 5) and the [^18^F]fluoride recovery ranged from 17.5% (Table 2, entry 16) to 74.7% (Table 2, entry 6). The [^18^F]fluoride recovery behaved in accordance with the EE. The highest elution efficiencies were achieved by using commercial non-carbonated 130 mg QMA cartridges (Table 2, entries 1–6). The PS-HCO_3_ cartridge behaved similarly to 130 mg QMA cartridges when comparing the amounts of the elution agent (Table 2, entries 7–12). Self-filled 10 mg QMA cartridges gave poorer results than the 130 mg QMA cartridges (Table 2, entries 13–18). Carbonated 46 mg QMA cartridges gave poor results (EE < 50%) regardless of the preconditioning of the cartridge (Table 2, entries 19–22). Increasing the molar amount of the elution agent increased the elution efficiency until a certain limit with every cartridge tested. The most significant differences in the EE were achieved with every cartridge (excluding carbonated 46 mg QMA cartridges) when the amount of the elution agent was increased from 24 μmol to 48 μmol. Cu(OTf)_2_ was always a better eluting agent than Cu(OTf)_2_(py)_4_, except when using a carbonated QMA cartridge.

**Table 2:** ^18^F-elution varying SPE cartridges, cartridge preconditioning, and the amount of Cu(OTf)_2_ or Cu(OTf)_2_(py)_4_

Entry (*n* = 3)	Cartridge	Cartridge pre-conditioning	Elution agent	Amount of the elution agent [μmol]	[^18^F]Fluoride recovery ± SD [%]	EE^a^ ± SD [%]
1	QMA 130 mg	0.5 M LiOTf, H_2_O	Cu(OTf)_2_(py)_4_	24	47.1 ± 9.7	52.1 ± 11.3
2	QMA 130 mg	0.5 M LiOTf, H_2_O	Cu(OTf)_2_(py)_4_	48	52.5 ± 5.0	58.5 ± 7.5
3	QMA 130 mg	0.5 M LiOTf, H_2_O	Cu(OTf)_2_(py)_4_	96	64.3 ± 10.1	68.1 ± 10.0
4	QMA 130 mg	0.5 M LiOTf, H_2_O	Cu(OTf)_2_	24	48.6 ± 0.6	53.4 ± 1.3
**5**	**QMA 130 mg**	**0.5 M LiOTf, H**_**2**_ **O**	**Cu(OTf)**_**2**_	**48**	**61.8 ± 9.1**	**69.2 ± 9.5**
6	QMA 130 mg	0.5 M LiOTf, H_2_O	Cu(OTf)_2_	96	65.8 ± 7.9	70.7 ± 7.1
**7**	**PS-HCO**_**3**_ **45 mg**	**H**_**2**_ **O**	**Cu(OTf)**_**2**_ **(py)**_**4**_	**12**	**24.2 ± 6.8**	**26.6 ± 7.3**
8	PS-HCO_3_ 45 mg	H_2_O	Cu(OTf)_2_(py)_4_	24	31.6 ± 9.1	35.3 ± 10.6
9	PS-HCO_3_ 45 mg	H_2_O	Cu(OTf)_2_(py)_4_	48	57.3 ± 4.9	63.5 ± 5.9
10	PS-HCO_3_ 45 mg	H_2_O	Cu(OTf)_2_	12	31.9 ± 18.8	35.6 ± 20.4
11	PS-HCO_3_ 45 mg	H_2_O	Cu(OTf)_2_	24	41.1 ± 7.5	49.1 ± 8.6
12	PS-HCO_3_ 45 mg	H_2_O	Cu(OTf)_2_	48	58.7 ± 9.0	64.5 ± 8.9
13	QMA 10 mg	0.5 M LiOTf, H_2_O	Cu(OTf)_2_(py)_4_	12	27.5 ± 8.3	31.4 ± 11.4
14	QMA 10 mg	0.5 M LiOTf, H_2_O	Cu(OTf)_2_(py)_4_	24	34.4 ± 8.8	40.5 ± 14.8
15	QMA 10 mg	0.5 M LiOTf, H_2_O	Cu(OTf)_2_(py)_4_	48	46.1 ± 9.9	52.9 ± 15.9
16	QMA 10 mg	0.5 M LiOTf, H_2_O	Cu(OTf)_2_	12	32.6 ± 13.4	38.8 ± 17.7
17	QMA 10 mg	0.5 M LiOTf, H_2_O	Cu(OTf)_2_	24	42.7 ± 6.3	50.4 ± 8.8
18	QMA 10 mg	0.5 M LiOTf, H_2_O	Cu(OTf)_2_	48	55.3 ± 3.6	62.8 ± 4.8
19	QMA carb 46 mg	H_2_O	Cu(OTf)_2_(py)_4_	48	38.7 ± 2.3	40.5 ± 3.1
20	QMA carb 46 mg	H_2_O	Cu(OTf)_2_	48	29.8 ± 5.8	32.1 ± 6.7
21	QMA carb 46 mg	0.5 M LiOTf, H_2_O	Cu(OTf)_2_(py)_4_	48	33.2 ± 5.4	35.1 ± 6.3
22	QMA carb 46 mg	0.5 M LiOTf, H_2_O	Cu(OTf)_2_	48	29.4 ± 11.3	31.4 ± 13.2

^a^*EE* elution efficiency. Entries with the lowest and the highest elution efficiencies are highlighted

### ^18^F-Radiolabelling

**Fig. 1:**
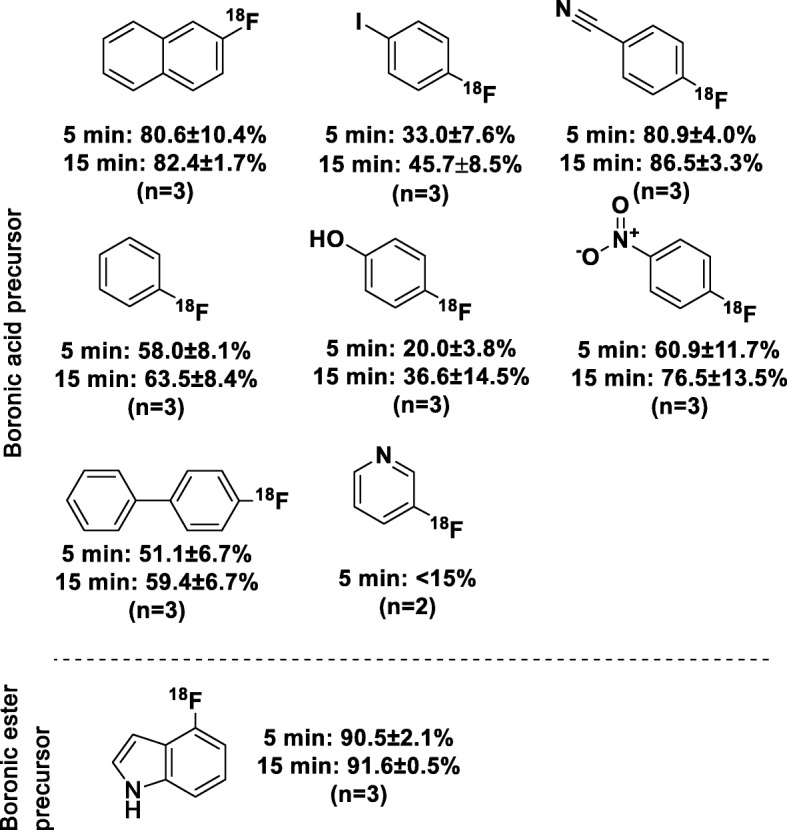
Products with reaction times and RCY (based on the HPLC analyses of the reaction solution) values of copper-mediated ^18^F-radiofluorination when using azeotropic drying free [^18^F]fluoride activation method and aryl boronic acid or aryl boronic ester precursors

Next, we tested if the optimized elution method (Table 2, entry 5) had an effect on the radiofluorination of aryl boronic acids, aryl boronic esters, and arylstannanes. Boronic acid precursors were used to synthetize 1-[^18^F]fluoro-4-iodobenzene, 4-[^18^F]fluorobiphenyl, 4-[^18^F]fluorophenol, [^18^F]fluorobenzene 4-[^18^F]fluorobenzonitrile, 1-[^18^F]fluoro-4-nitrobenzene, 3-[^18^F]fluoropyridine, 2-[^18^F]fluoronaphthalene; boronic ester precursor was used to synthetize 4-[^18^F]fluoroindole and trimethylstannyl precursors were used to synthetize [^18^F]NS12137 and [^18^F]CFT. Even though our previous results (Lahdenpohja et al. 2019) showed, that a 2 min reaction at 120 °C is enough for optimal RCY (based on the HPLC analyses of the reaction solution), here we used 5 to 15 min reaction times because most related works have used 20 min reactions. Results from the ^18^F-labelling reactions are presented in Fig. 1. Different model molecules were produced up to 91.6% RCY (based on the HPLC analyses of the reaction solution). The lowest RCY (based on the HPLC analyses of the reaction solution) was achieved with 3-[^18^F]fluoropyridine and the highest with 4-[^18^F]fluoroindole.

**Fig. 2:**
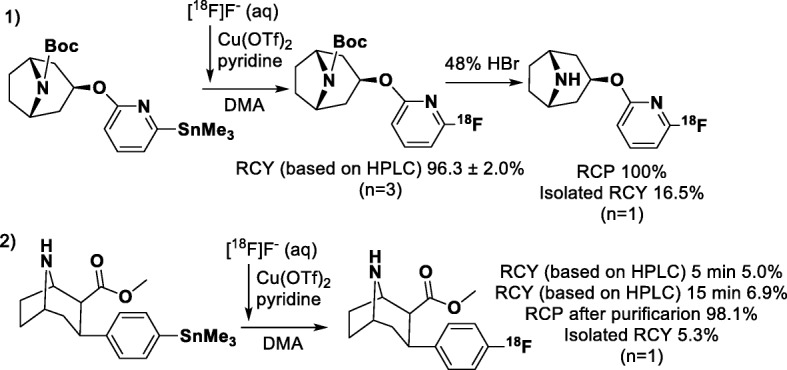
Copper-mediated radiofluorination of 1) [^18^F]NS12137 with 5 min reaction and 5 min acid hydrolysis and purification, and 2) [^18^F]CFT with 5 to 15 min reaction and purification. Reaction conditions for both: 1 eq precursor, 5 eq Cu(OTf)_2_, 75 eq pyridine, 120 °C

Finally, as proof-of concept, we synthetized [^18^F]NS12137 and [^18^F]CFT (Fig. 2) with optimized elution conditions (Table 2, entry 5). The [^18^F]NS12137 intermediate was achieved with 96.3 ± 2.0% RCY (based on the HPLC analyses of the reaction solution) after a 5 min reaction at 120 °C. Incorporation of the [^18^F]fluoride was 37.7% according to the SPE and after the hydrolysis and semi-preparative HPLC purification, [^18^F]NS12137 was produced in 16.5% RCY and 100% RCP in 98 min synthesis time (*n* = 1). [^18^F]CFT was achieved in 6.9% RCY (based on the HPLC analyses of the reaction solution) after a 15 min reaction at 120 °C. After the semi-preparative purification, [^18^F]CFT was produced in 5.3% RCY and 98.1% RCP in an overall synthesis time of 64 min (n = 1). According to the ICP-MS analysis of the final product fractions, the amount of Cu-63 residue was 3.9 μg in [^18^F]NS12137 and 46.0 μg in [^18^F]CFT. These amounts are under the limits considered toxic according to ICH Q3D (International Council for Harmonisation of Technical Requirements for Pharmaceuticals for Human Use n.d.).

## Discussion

### [^18^F]fluoride processing

A key step in the ^18^F-radiolabelling reactions is the drying of [^18^F]fluoride to make it more reactive, i.e., a better nucleophile. Conventionally used azeotropic distillation with MeCN is time consuming and here we have replaced it with SPE by using anion exchange cartridges. Lately, the use of SPE for the drying of [^18^F]fluoride has also been a widely studied method in copper-mediated ^18^F-radiofluorination, but typically these methods have still utilized additional evaporation steps (Zischler et al. 2017) or use of additional, sometimes toxic reagents (Antuganov et al. 2019; Zhang et al. 2019). Herein, we have described a simple method, which includes the use of Cu(OTf)_2_ or Cu(OTf)_2_(py)_4_ for the elution of [^18^F]fluoride, and is suitable for automation and clinical production of radiopharmaceuticals.

Our preliminary studies showed that the preconditioning of the 130 mg QMA cartridges and carbonated 46 mg QMA cartridges has a significant effect on [^18^F]fluoride recovery and elution efficiency (see Table 1). LiOTf was found to be a superior preconditioning agent providing up to 70% [^18^F]fluoride recovery when using non-carbonated 130 mg QMA cartridges. Besides, the addition of LiOTf to the elution solution enhanced the elution. This method proposes that the anions are effectively changed from chlorine to triflate or from carbonate to triflate inside the cartridge, and this improves the elution of [^18^F]fluoride. Similar enhanced elution was not detected when using PS-HCO_3_ cartridges or self-filled 10 mg QMA-cartridges. PS-HCO_3_ cartridges might have needed a higher amount of LiOTf in the preconditioning step to replace the bicarbonate. In self-filled 10 mg QMA cartridges, the amount of the anion exchange resin is minimized, which can be detected as reduced trapping efficiency of the cartridge, i.e., leakage of the fluoride during trapping and washing. This is also the reason for large difference in elution efficiency and (Ross et al. 2007)fluoride recovery (Table 1, entries 1–4). Minimized resin mass reduces the need for elution enhancer, such as LiOTf as preconditioning agent or elution agent. When using Na_2_SO_4_ solution for the preconditioning, elution efficiency was almost 0%. Such a drastic decrease in the elution compared to the standard level may likely be caused by deactivation of the eluting agent by formation of an inactive Cu(SO_2_)_2_(py)_4_ complex (Mossine et al. 2017).

Cu(OTf)_2_ was, except with carbonated cartridge, a better elution agent than Cu(OTf)_2_(py)_4_. We propose that this result is caused by the steric hindrance of Cu(OTf)_2_(py)_4_ during the coordination of [^18^F]fluoride. We suggest that [^18^F]fluoride coordinates to the copper complex in the anion exchange resin as previously suggested with alcohol-enhanced copper-mediated radiofluorination (Zarrad et al. 2017). When using large amounts of the copper-complex, the difference in the EE between these two elution agents became discreet. The optimal conditions were chosen to be 48 μmol of Cu(OTf)_2_ and non-carbonated 130 mg QMA cartridge even though higher amount of copper complex slightly improved the [^18^F]fluoride recovery. Additionally, large amounts of copper in the reaction solution might complicate the purification of the final product which supports our choice of optimal amount. Also, PS-HCO_3_ cartridge behaved similarly to non-carbonated 130 mg QMA cartridge, however, the QMA cartridge was chosen because of being one of the most standard anion exchange cartridges used in radiopharmaceutical chemistry laboratories. High deviations in EEs were observed with both elution agents, Cu(OTf)_2_ and Cu(OTf)_2_(py)_4_, as presented in Table 2. We suggest that the EE is very sensitive to the preconditioning speed. According to the results with non-carbonated 130 mg QMA cartridges (Table 1, entries 5, 8), the EE was higher with LiOTf as an additional elution agent than LiOTf as a preconditioning agent, this might be caused by negligent preconditioning.

### ^18^F-Radiolabelling

Using SPE for the drying of [^18^F]fluoride did not have a negative effect on the subsequent labelling reaction and negative effects of the azeotropic drying can be avoided. With time consuming azeotropic drying, some of the [^18^F]fluoride is typically attached to the glass vial. In the present procedure in the synthesis of [^18^F]NS12137 with SPE, the incorporation of the [^18^F]fluoride is improved with approximately 10% when comparing to the azeotropic distillation method (Lahdenpohja et al. 2019). In turn, when drying the [^18^F]fluoride in a traditional way, the vial is usually still warm when the subsequent reaction is started which can, in some cases, promote the reaction and shorten the reaction time slightly.

With [^18^F]CFT and the model molecules synthetized in this study, we noticed that increasing the reaction time from 5 to 15 min had only a minor increase in the RCY. Additionally, free [^18^F]fluoride was detected only in minimal amounts in all of the analyzed crude reaction mixtures according to HPLC analysis. This outcome implies that all the radioactivity in the reaction solution is in an active form. With [^18^F]NS12137, we observed good incorporation of the [^18^F]fluoride according to SPE. The lowest RCY was achieved with 3-[^18^F]fluoropyridine. Unsuccessful reaction is likely caused by the coordination of the precursor molecule to Cu^2+^ instead of with pyridine. In the setup presented here, pyridine-3-boronic acid might have coordinated to the copper complex, because the elution solution containing the copper complex Cu(OTf)_2_ was added to the reaction vial containing both pyridine-3-boronic acid and additional pyridine. With this method, the substrate scope is wide, but the order of the reagent additions must be taken into account, especially in the case of pyridine-containing structures.

## Conclusions

In conclusion, we have described the fast and efficient copper-mediated ^18^F-fluorination of arylstannanes and aryl boronic acids by using SPE for the drying of the [^18^F]fluoride. This new method does not require azeotropic drying of [^18^F]fluoride or any other evaporation steps, which makes the process more simple and straightforward. We demonstrated that alterations in the [^18^F]fluoride elution techniques, like preconditioning of the cartridge, amount of the eluting agent, and the elution speed, can have significant effects on the [^18^F]fluoride recovery and thus on the RCY of the final tracer. We successfully applied the optimized elution method with Cu(OTf)_2_ to the semi-automated production of model molecules with high RCY and the clinically relevant molecules, [^18^F]NS12137 and [^18^F]CFT, in moderate RCY. We anticipate these improved [^18^F]fluoride elution techniques will supplant the traditional azeotropic drying of [^18^F]fluoride in clinical radiopharmaceutical production and eventually, increase the achievable RCYs. Our on-going work is concentrated on implementing the method on fully automated synthesis platform and ultimately in clinical radiopharmaceutical production.

## Additional file

**Additional file 1.** More detailed description of analytical HPLC and SPE methods as well as radiochemistry studies are presented in the Additional file. (DOCX 1151 kb)

## Data Availability

All data generated or analyzed during this study are included in this published article and its supplementary information files.

## Abbreviations

A_m_: Molar activity
DMA: Dimethylacetamide
EE: Elution efficiency
EOB: End of bombardment
EOS: End of the synthesis
HPLC: High pressure liquid chromatography
PET: Positron emission tomography
QMA: Quaternary methyl ammonium
RCP: Radiochemical purity
RCY: Radiochemical yield
SPE: Solid phase extraction
THF: Tetrahydrofuran
