# Supplementary material for: Fast and efficient copper-mediated 18F-fluorination of arylstannanes, aryl boronic acids, and aryl boronic esters without azeotropic drying
Source: EJNMMI Radiopharm Chem. 2019 Oct 16;4:28. doi: 10.1186/s41181-019-0079-y (PMC6795642; doi:10.1186/s41181-019-0079-y)
Supplement: Supplementary file 1 — Additional file 1. More detailed description of analytical HPLC and SPE methods as well as radiochemistry studies are presented in the Additional file. (DOCX 1151 kb) [file 41181_2019_79_MOESM1_ESM.docx]

# SUPPORTING INFORMATION

# Fast and efficient copper-mediated ^18^F-fluorination of arylstannanes, aryl boronic acids, and aryl boronic esters without azeotropic drying

Salla Lahdenpohja^1^, Noora Rajala^1^, Johan Rajander^2^, Anna K. Kirjavainen^1^*

^1^Radiopharmaceutical Chemistry Laboratory, Turku PET Centre, University of Turku, Turku, Finland

^2^Accelerator Laboratory, Turku PET Centre, Åbo Akademi University, Turku, Finland

*Corresponding author

# Methods

## General

Unless otherwise stated, all of the reagents and solvents were used as received from commercial suppliers without further purification. The [^18^F]NS12137 precursor (*exo*-*tert*-butyl-3-[(6-trimethylstannyl-2-pyridyl)oxy]-8-azabicyclo-[3.2.1]octane-8-carboxylate) and the reference compounds *exo*-*tert*-butyl-3-[(6-fluoro-2-pyridyl)oxy]-8-azabicyclo-[3.2.1]octane-8-carboxylate and *exo*-3-[(6-[^18^F]fluoro-2-pyridyl)oxy]8-azabicyclo[3.2.1]octane were obtained from DanPET AB (Malmö, Sweden, for characterization see (Lahdenpohja et al. 2019)). The [^18^F]CFT precursor and the reference compounds were synthesised as previously reported. (Ametamey et al. 1995) Boronic acid precursors were used to synthetize 1-[^18^F]fluoro-4-iodobenzene, 4-[^18^F]fluorobiphenyl, 4-[^18^F]fluorophenol, [^18^F]fluorobenzene 4‑[^18^F]fluorobenzonitrile, 1‑[^18^F]fluoro-4-nitrobenzene, 3-[^18^F]fluoropyridine, 2‑[^18^F]fluoronaphthalene; boronic ester precursor was used to synthetize 4-[^18^F]fluoroindole and trimethylstannyl precursors were used to synthetize [^18^F]NS12137 and [^18^F]CFT.

## Solid phase extraction

Anion exchange cartridges were purchased as follows: 1) Sep-Pak Accell Plus QMA Carbonate Plus Light Cartridge, 46 mg (Waters Corp., Milford, MA, USA) 2) Sep-Pak Accell Plus QMA Plus Light Cartridge, 130 mg (Waters Corp.) and 3) Chromafix PS-HCO_3_ 45 mg (Synthra, Hamburg, Germany). Also 10 mg QMA cartridges were used, they were hand made using the packing material of the QMA Plus Light Cartridge. For the SPE cartridge preconditioning, 1) 20 mL H_2_O or 2) 10 mL 0.5 M LiOTf and 20 mL H_2_O or 3) 10 mL 0.5 M Na_2_SO_4_ and 20 mL H_2_O.

## HPLC methods

Semi-preparative radio-HPLC purification was performed with a Jasco PU-2089 Plus (JASCO Europe s.r.l., Cremella, Italy) preparative HPLC pump. Purification of [^18^F]NS12137 (Kirjavainen et al. 2018) was carried out as previously described. For purification of [^18^F]CFT 7 mM KH_2_PO_4_ (A) and MeCN (B) were used as eluents with following method: 0 to 5 min 100 % A, 5 to 20 min 100 to 20 %. A Gemini NX C18 column (110 Å, 5 µm, 10 × 250 mm; Phenomenex, Milford, MA USA) was used with a flow rate of 5.0 ml/min.

Analytical radio-HPLC was carried out with a VWR Hitachi L-2130 HPLC pump (VWR Hitachi, VWR International GmbH, Darmstadt, Germany) equipped with a VWR Hitachi L-2400 UV-absorption detector and a 2 × 2-inch NaI radioactivity detector. Following columns were used for analysis: Luna C5 column (100 Å, 5 μm, 4.6 × 150 mm, Phenomenex) and Gemini C18 column (110 Å, 5 µm, 4.6 × 250 mm; Phenomenex). Analytical methods used are listed in the Supp. Table 1.

**Supplementary Table 1**. Analytical HPLC methods.

| **[^18^F]NS12137 intermediate** | |  | **[^18^F]NS12137** | |  | **[^18^F]CFT** | |
| --- | --- | --- | --- | --- | --- | --- | --- |
| Column: Luna C5 | |  | Column: Gemini C18 | |  | Column: Gemini C18 | |
| 1.5 ml/min, λ = 230 nm | |  | 1.5 ml/min, λ = 230 nm | |  | 1.5 ml/min, λ = 215 nm | |
| A: H_2_O+0.1%TFA  B: CH_3_CN+0.1% TFA | |  | A: 7 mM KH_2_PO_4_  B: CH_3_CN | |  | A: 7 mM KH_2_PO_4_  B: CH_3_CN | |
| 0 – 3 min | 80% A |  | 0 – 5 min | 100% A |  | 0 – 8 min | 100% A |
| 3 – 3.5 min | 80 – 40% A |  | 5 – 5.1 min | 100 – 92% A |  | 8 – 16 min | 100 – 50% A |
| 3.5 – 15 min | 40% A |  | 5.1 – 20 min | 92% A |  | 16 – 22 min | 50% A |

| **1-[^18^F]fluoro-4‑iodobenzene, 4‑[^18^F]fluorobiphenyl,**  **1‑[^18^F]fluoro-4‑nitrobenzene** | |  | **4-[^18^F]fluorophenol,**  **4‑[^18^F]fluorobenzene, 4‑[^18^F]fluorobenzonitrile, 3‑[^18^F]fluoropyridine, 2‑[^18^F]fluoronaphthalene, 4‑[^18^F]fluoroindole** | |
| --- | --- | --- | --- | --- |
| Column: Luna C5 | |  | Column: Luna C5 | |
| 1.5 ml/min, λ = 254 nm | |  | 1.5 ml/min, λ = 254 nm | |
| A: H_2_O+0.1%TFA  B: CH_3_CN+0.1% TFA | |  | A: H_2_O+0.1%TFA  B: CH_3_CN+0.1% TFA | |
| 0 – 3 min | 80% A |  | 0 – 3 min | 80% A |
| 3 – 3.5 min | 80 – 40% A |  | 3 – 3.5 min | 80 – 20% A |
| 3.5 – 15 min | 40% A |  | 3.5 – 15 min | 20% A |

## Analysis of copper content

## Levels of copper in [^18^F]NS12137 and [^18^F]CFT were analysed with inductively coupled plasma mass spectrometry (ICP-MS, PerkinElmer, Elan DRC Plus). Commercial multielement standard was used for the instrument calibration.

## Radiochemistry studies

[^18^F]Fluoride elution and the radiolabelling reactions were performed in hot cells with a remote-controlled synthesis device built in-house. The device used for the ^18^F-fluoride elution has been built so, that the SPE cartridges were loaded and washed from the female luer side and eluted from the male luer side to maximize the [^18^F]fluoride recovery.

Aqueous [^18^F]fluoride was produced by irradiating oxygen‐18 enriched water (3 mL) as previously reported (Savisto et al. 2017). The initial amount of [^18^F]fluoride was 60 – 300 MBq for the [^18^F]fluoride elution studies and 120 - 600 MBq for the radiolabelling studies. For [^18^F]NS12137 and [^18^F]CFT the initial amount of [^18^F]fluoride was 5 GBq.

Initial [^18^F]fluoride from the cyclotron was diluted with water to the total volume of 8 to 10 mL and loaded to a SPE cartridge. After the loading, activity of the SPE cartridge and the waste bottle were measured. The cartridge was washed with dimethylacetamide (DMA, 5 mL) and activity of the cartridge and the waste bottle were again measured. [^18^F]Fluoride was eluted with Cu(OTf)_2_ or Cu(OTf)_2_(py)_4_ in DMA (0.5 mL). The amount of the copper-complex was varied between 12 and 96 μmol. In the preliminary test, LiOTf (24 μmol) was added to the elution solution. The cartridge was washed with additional 0.5 mL of DMA, and activity of the eluted [^18^F]fluoride fraction and the SPE cartridge were measured. Elution efficiency (EE) was calculated by dividing the activity of the eluted [^18^F]fluoride fraction by the sum of the activity of the eluted [^18^F]fluoride fraction and the activity remaining in the SPE cartridge. [^18^F]Fluoride recovery was calculated by dividing the activity of the eluted [^18^F]fluoride fraction by the sum of the activity of the SPE cartridge and the waste bottle after loading of the cartridge. The EE and the [^18^F]fluoride recovery are non-decay corrected because the [^18^F]fluoride loading and elution and the radioactivity measurements were completed within 5 min.

In the radiolabelling test, [^18^F]fluoride was eluted straight to a 5 mL v-vial containing the labelling precursor (8 – 10 μmol) and pyridine (50µL) in DMA (50 µL). The reaction solution was heated at 120 ˚C for 15 min under ambient air. Samples for analytical HPLC were collected during the reaction at 5 and 15 min. In the case of [^18^F]NS12137, previously published deprotection method was followed. (Kirjavainen et al. 2018; Lahdenpohja et al. 2019) After 5 min labelling reaction, reaction solvent was first exchanged from DMA to THF with C18 cartridge (Waters Corp., preconditioned with 5 ml EtOH and 10 ml H_2_O), THF was evaporated and the deprotection was carried out in 48% HBr in 5 min. After the deprotection, the reaction mixture was diluted with water and purified with semi-preparative HPLC. In the case of [^18^F]CFT, reaction mixture was diluted with water after 15 min labelling reaction and purified with semi-preparative HPLC.

Each radioactive product signal was identified by comparing it to the corresponding UV signal of the reference material. All reported RCY values are decay-corrected to the end of the bombardment (EOB) and are expressed as mean ± standard deviation. The radiochemical purity (RCP) values have been determined from the radio-HPLC. No radioactivity retained in the HPLC column after the run.

# HPLC chromatograms

**[^18^F]NS12137 intermediate**


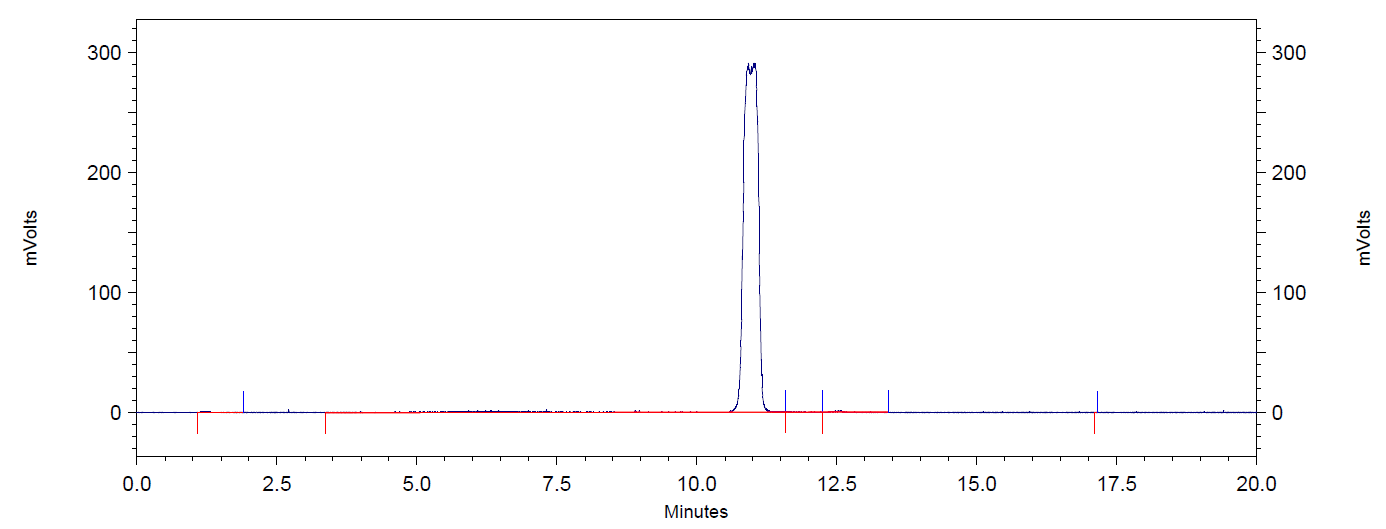

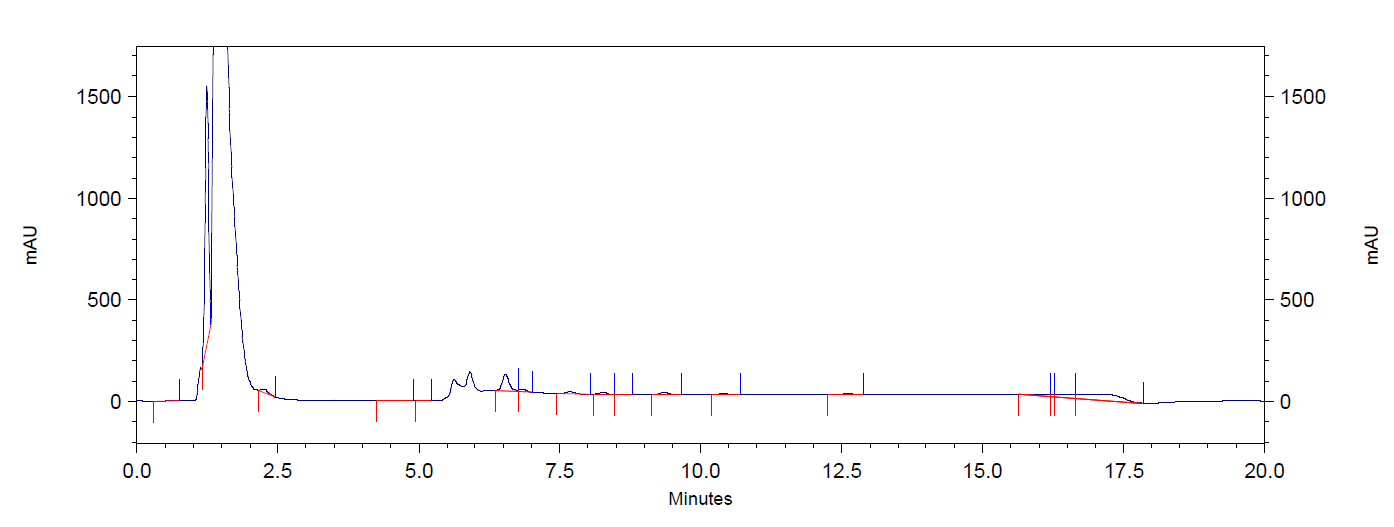


Radioactivity

(Double peak caused by high radioactivity concentration in the detector)

UV


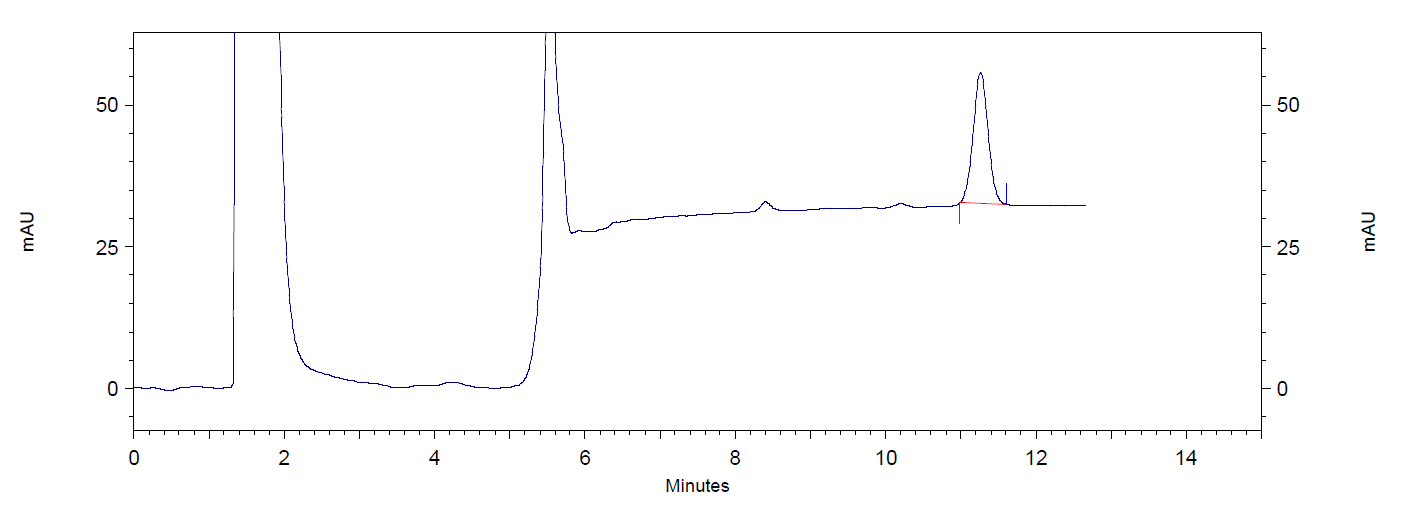


Reference UV

**[^18^F]NS12137 – purified product**


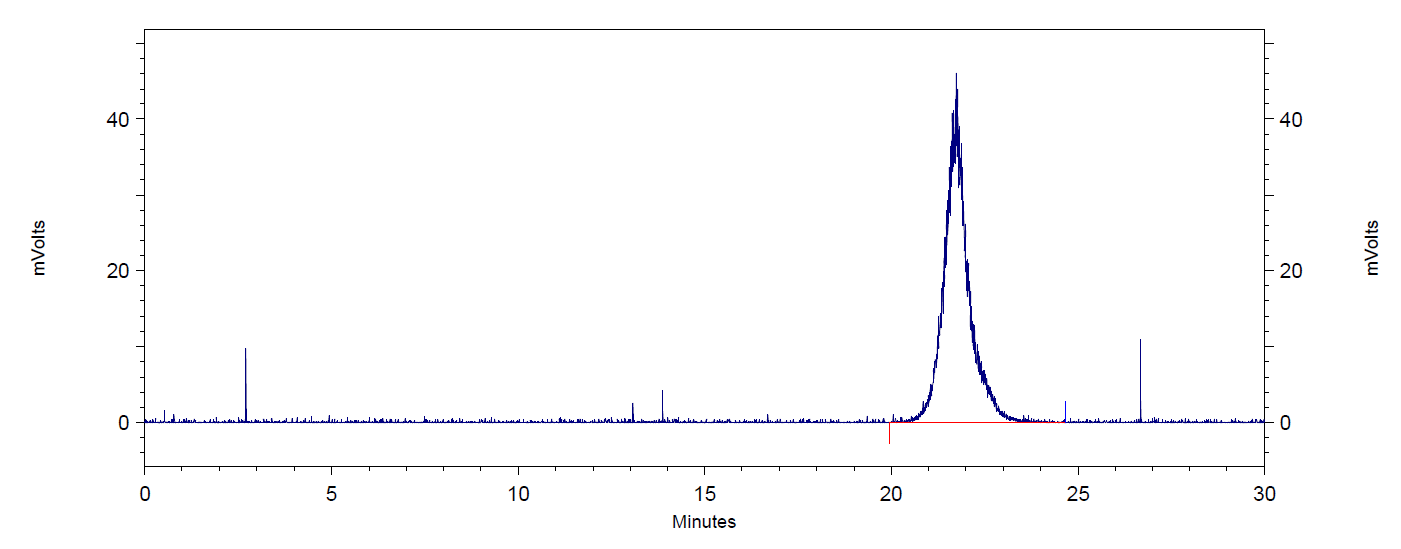


Radioactivity


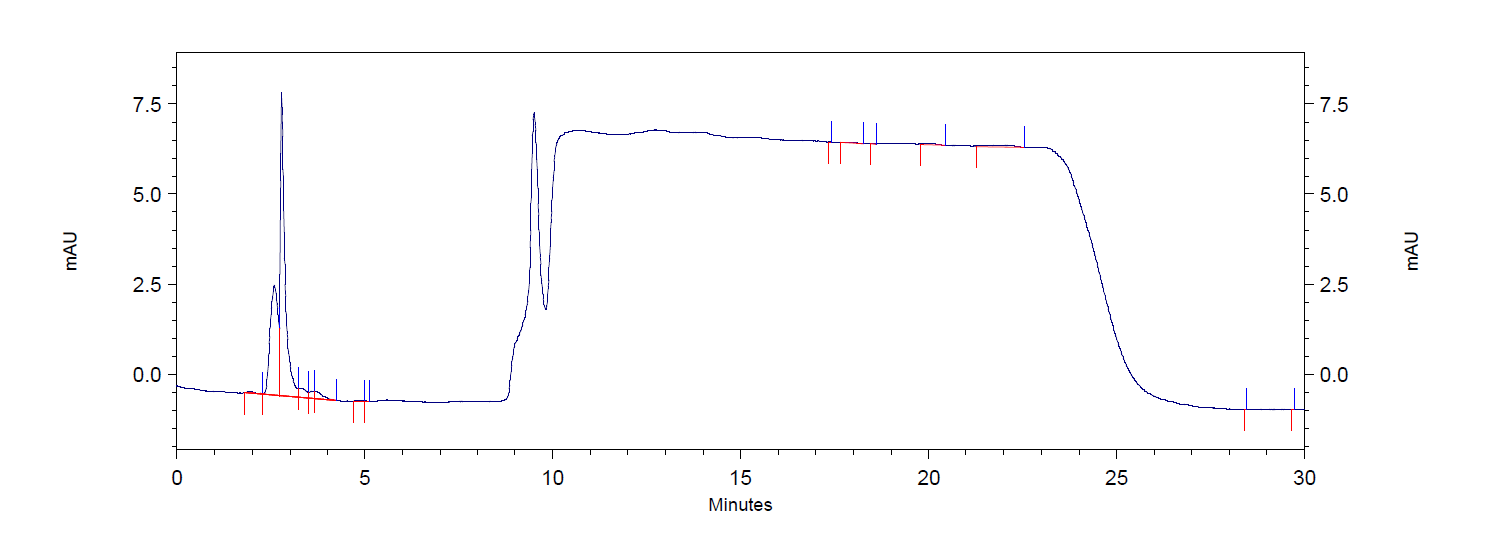


UV

Reference UV


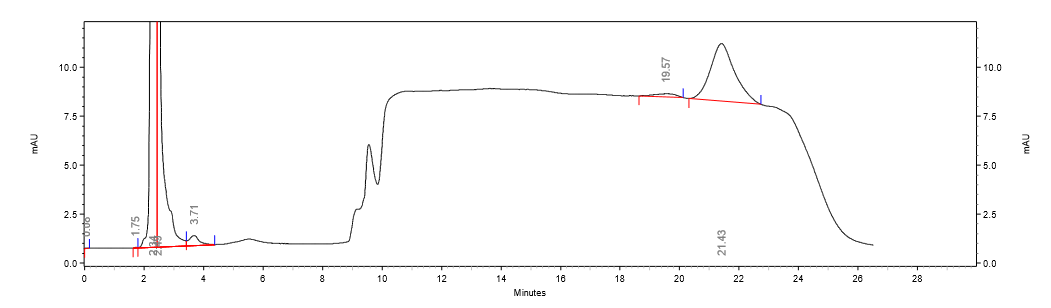


**[^18^F]CFT**


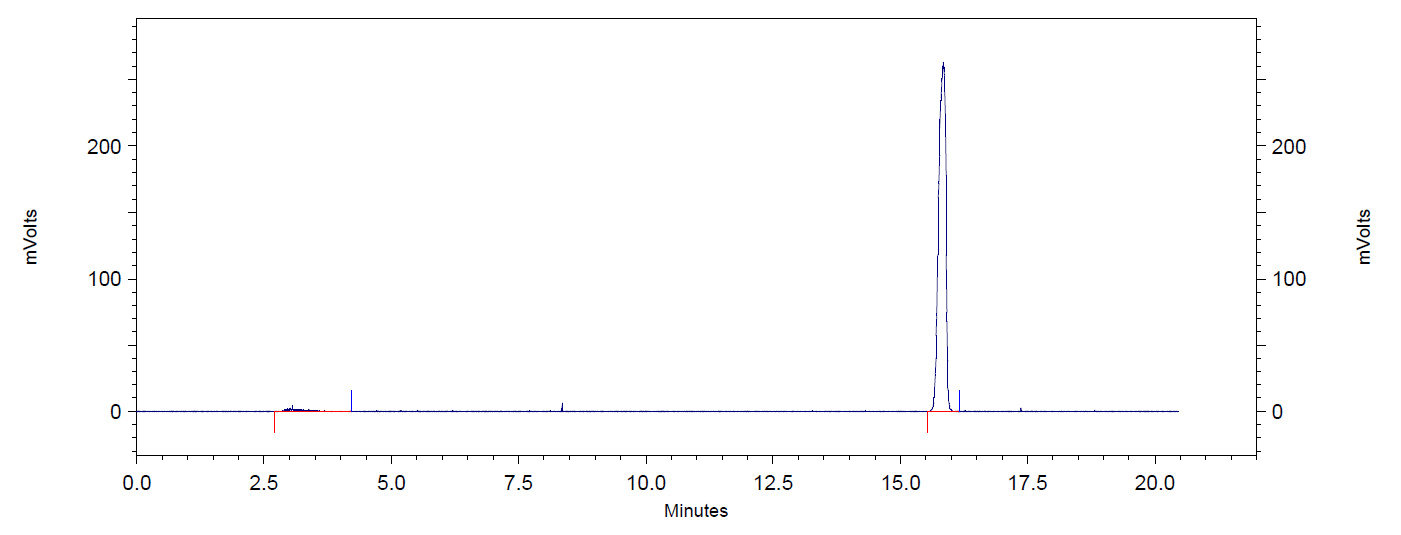

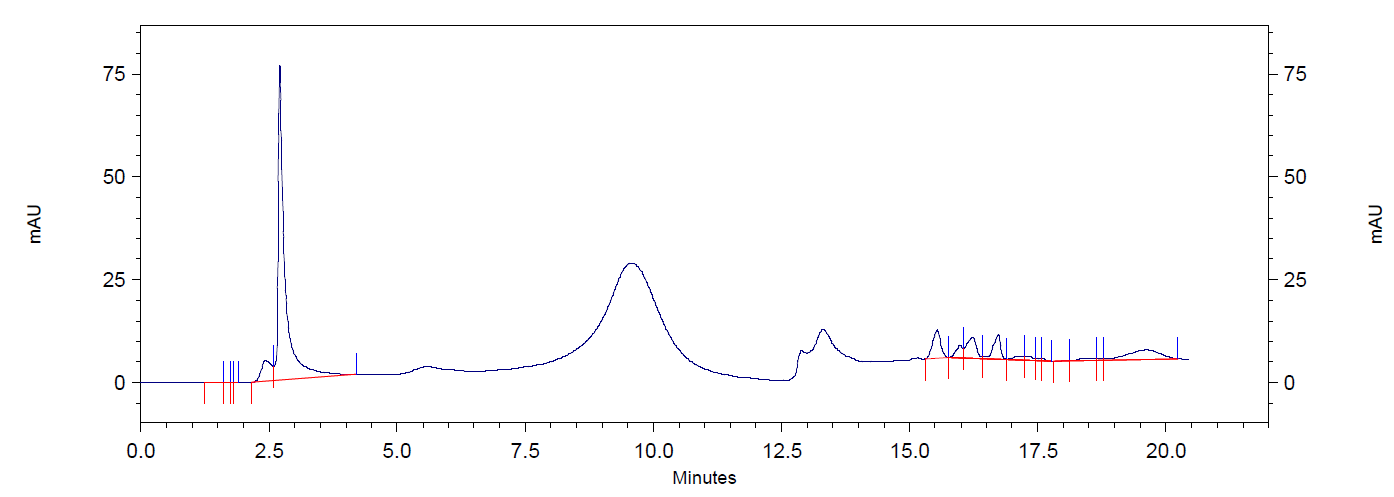


UV

Radioactivity


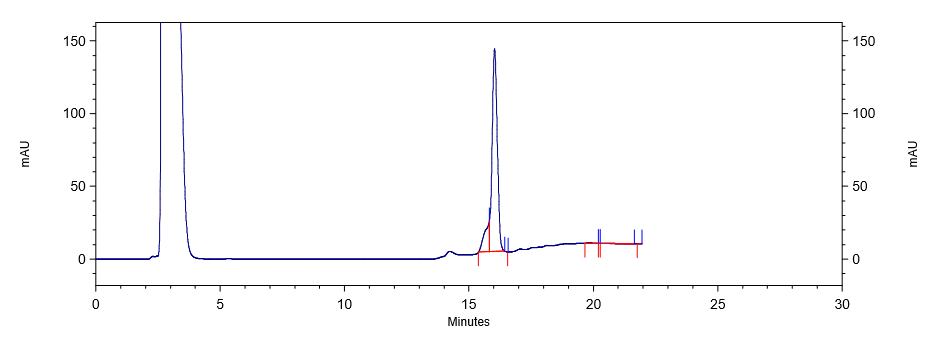


Reference UV

**1-[^18^F]fluoro-4‑iodobenzene**


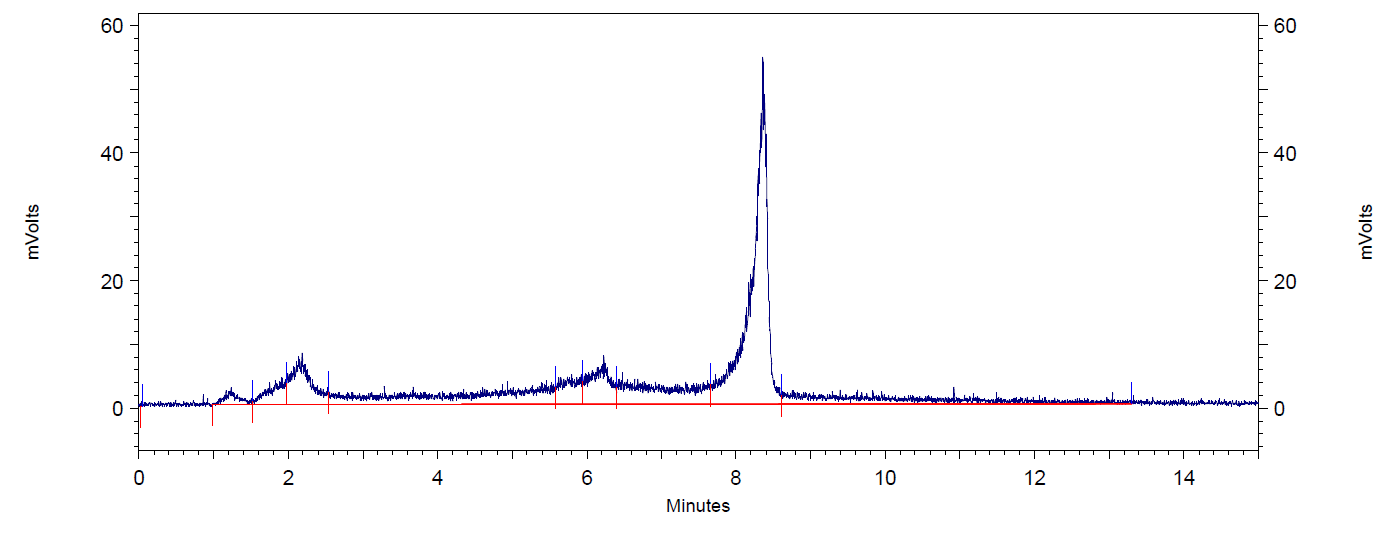


Radioactivity


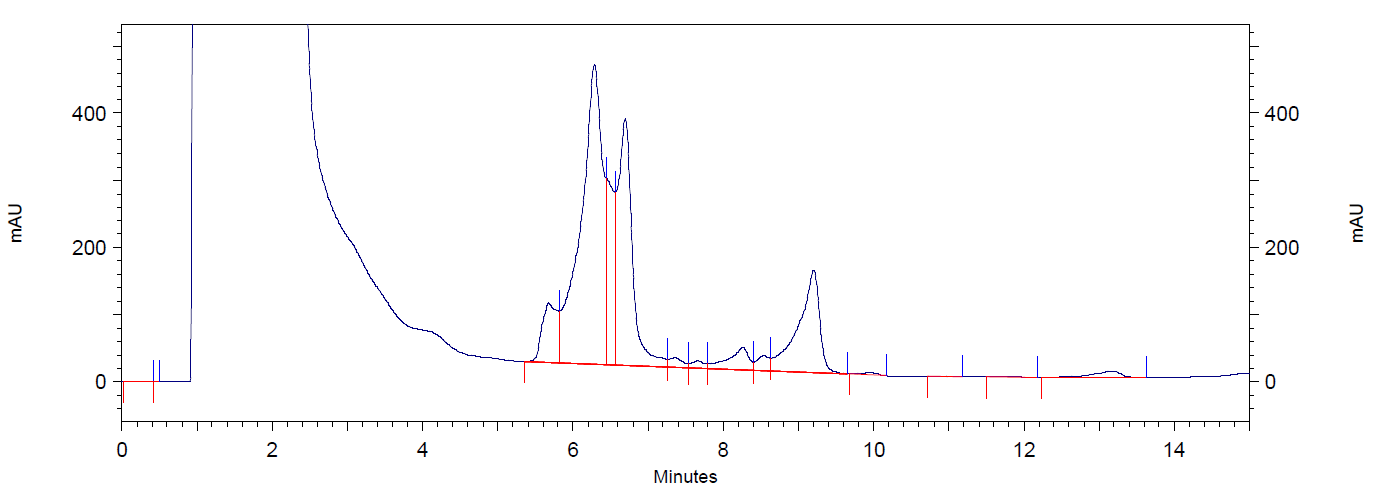


UV


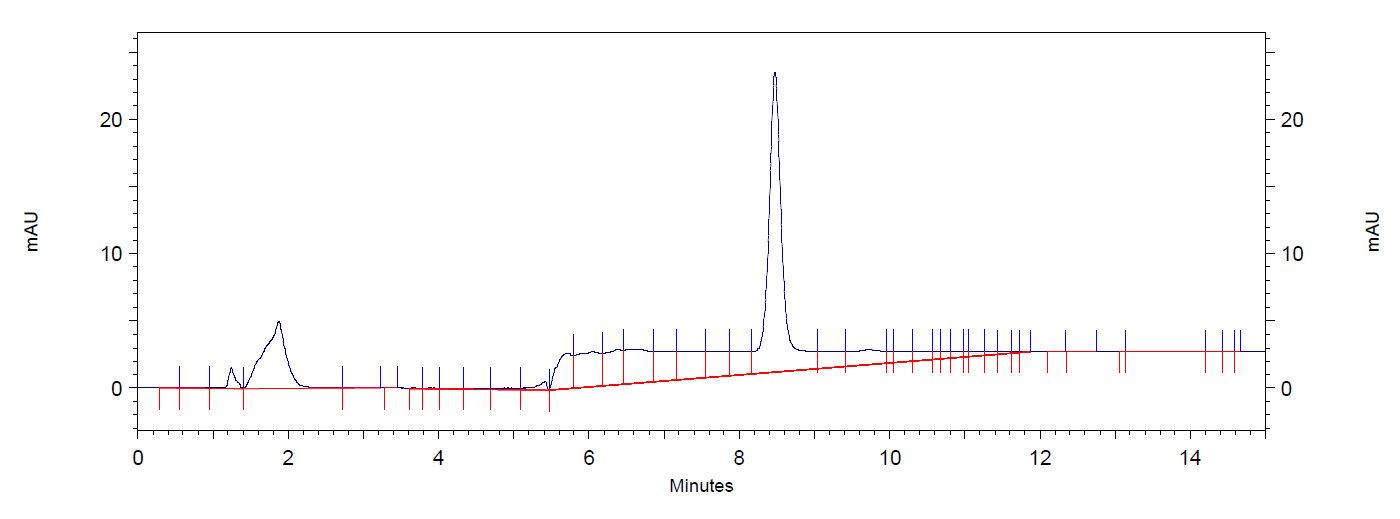


Reference UV

**4‑[^18^F]fluorobiphenyl**


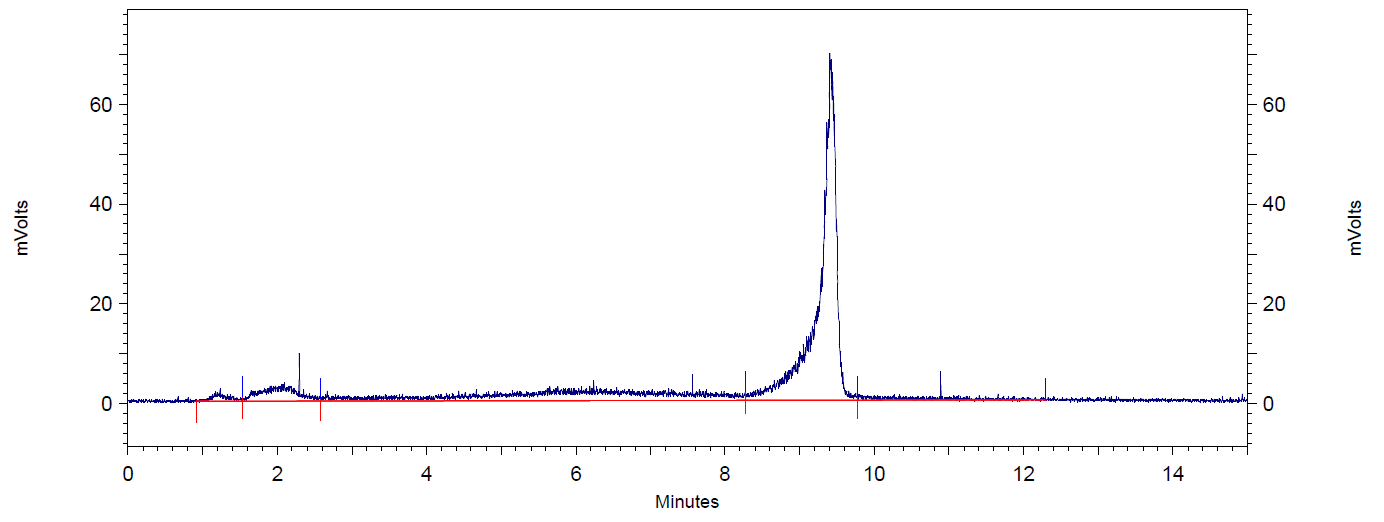

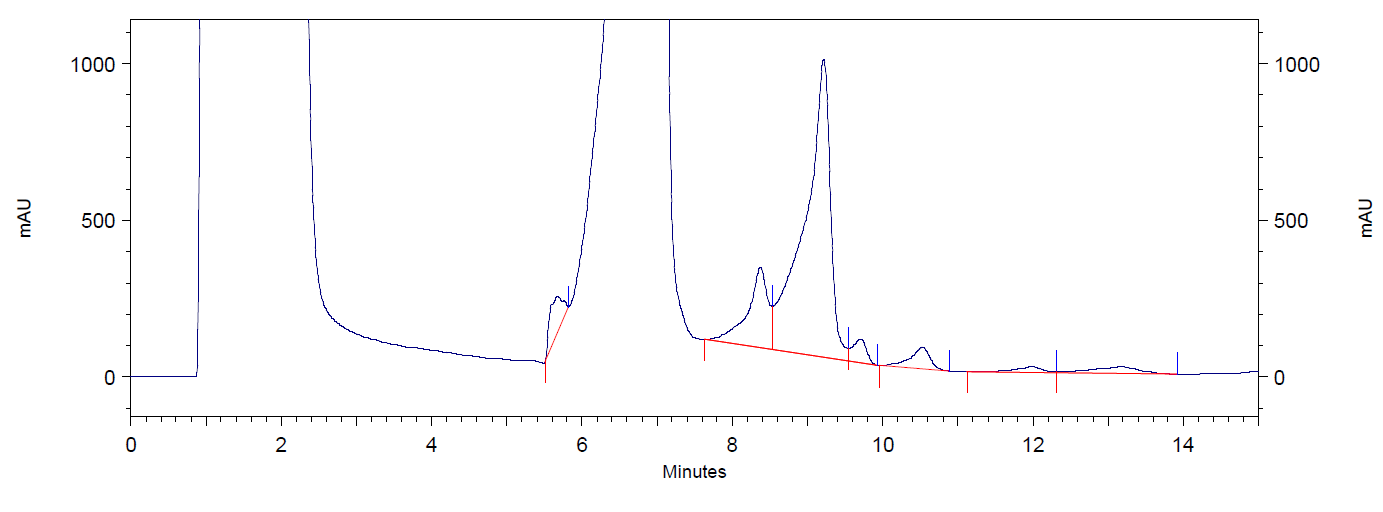


UV

Radioactivity


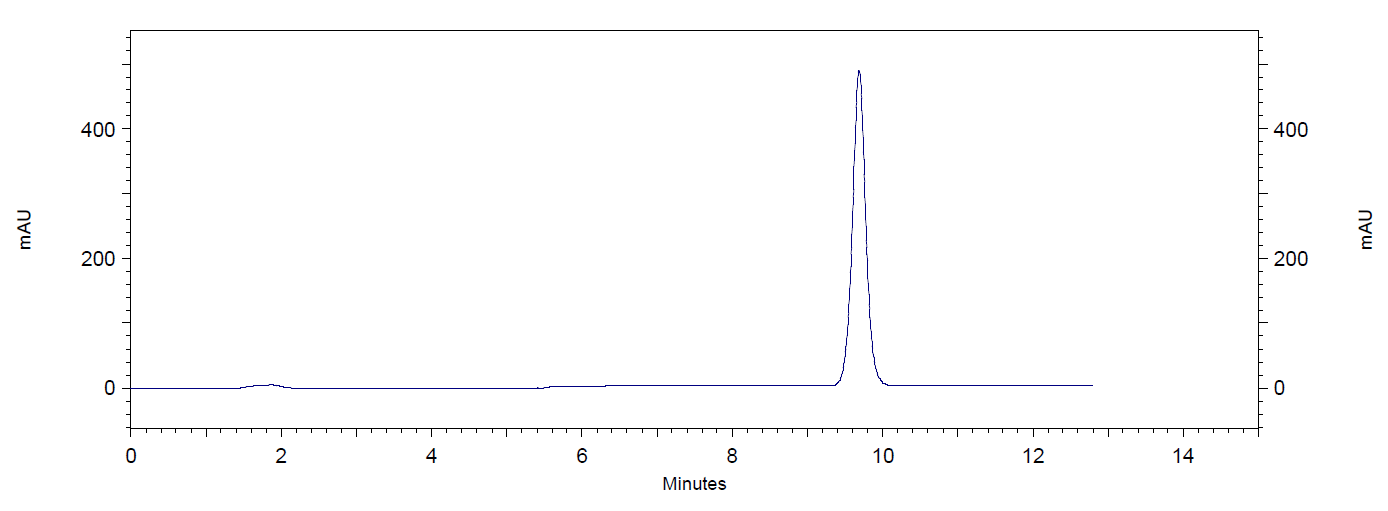


Reference UV

**1‑[^18^F]fluoro-4‑nitrobenzene**


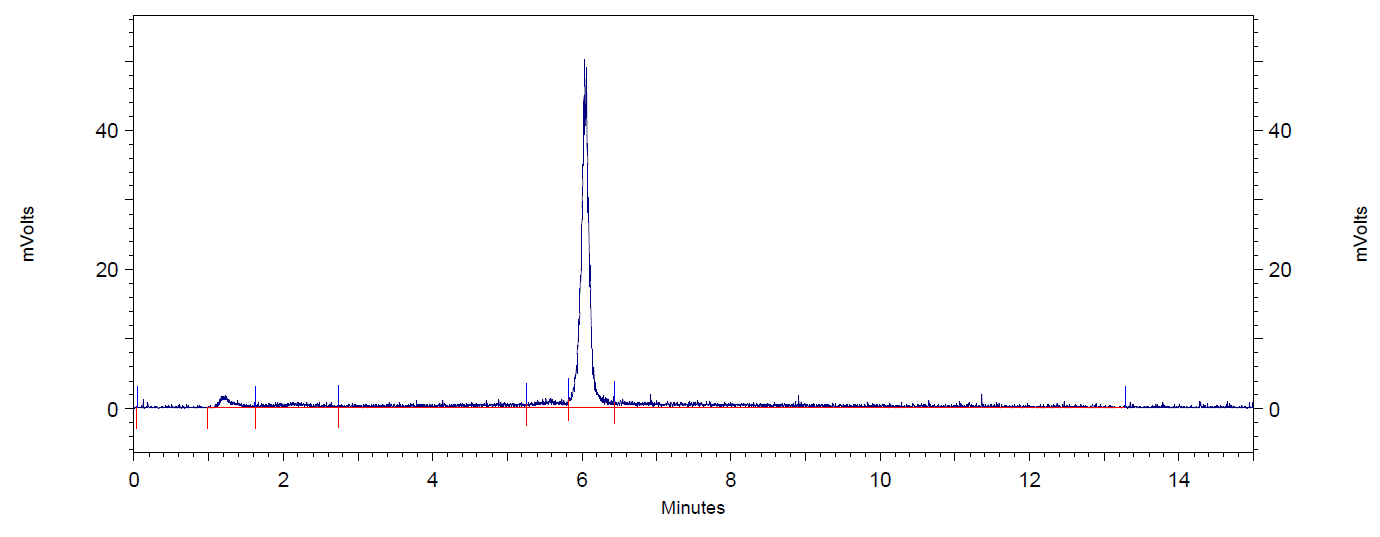

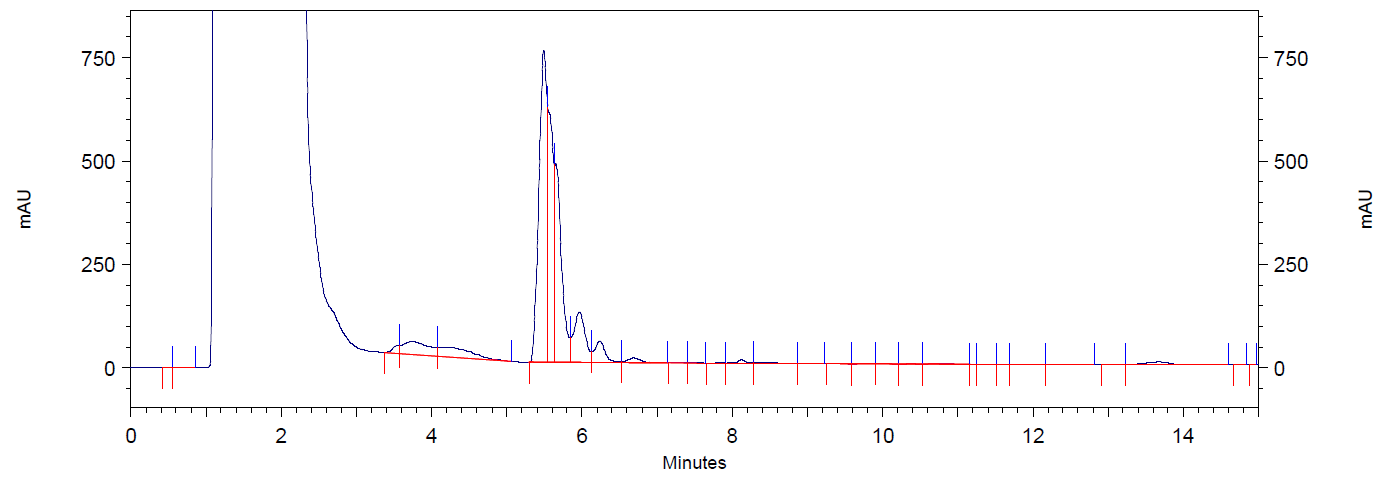


UV

Radioactivity


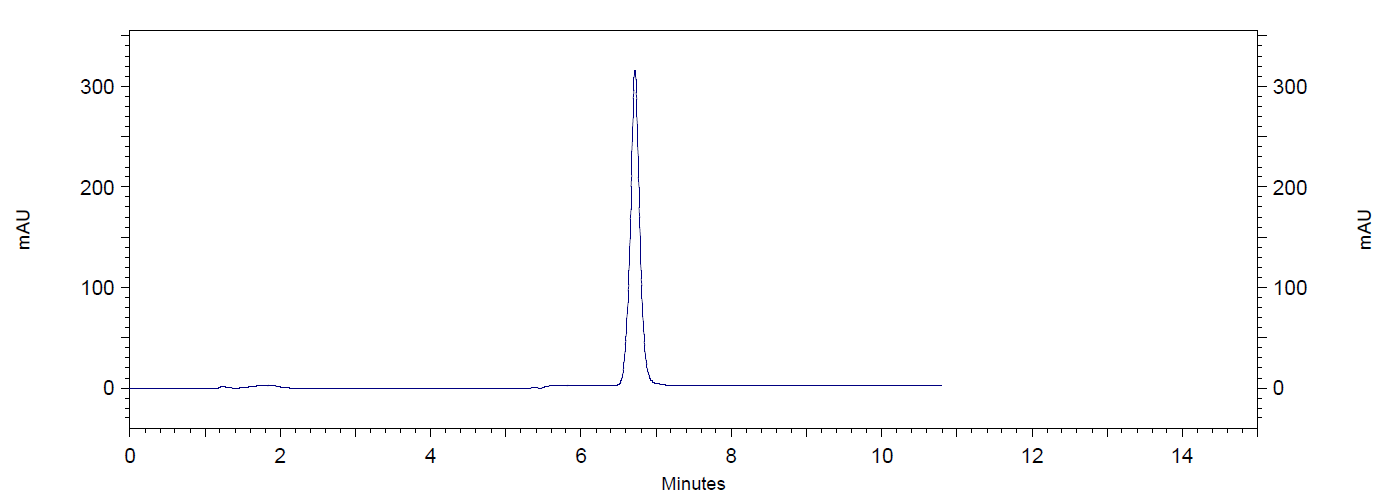


Reference UV

**4-[^18^F]fluorophenol**


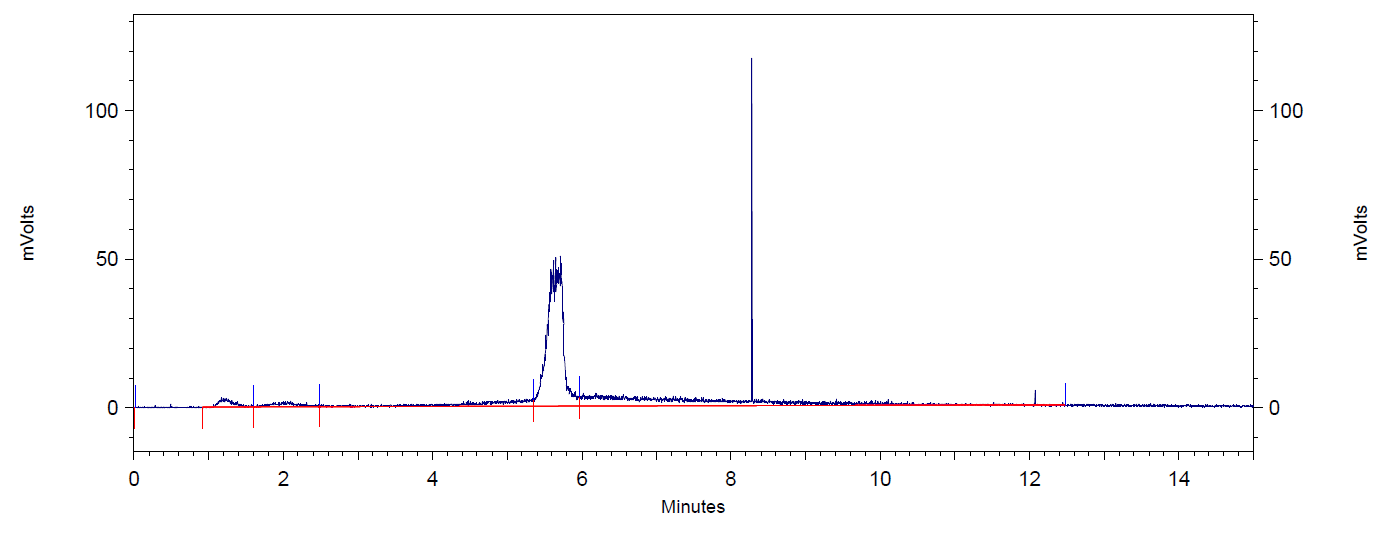

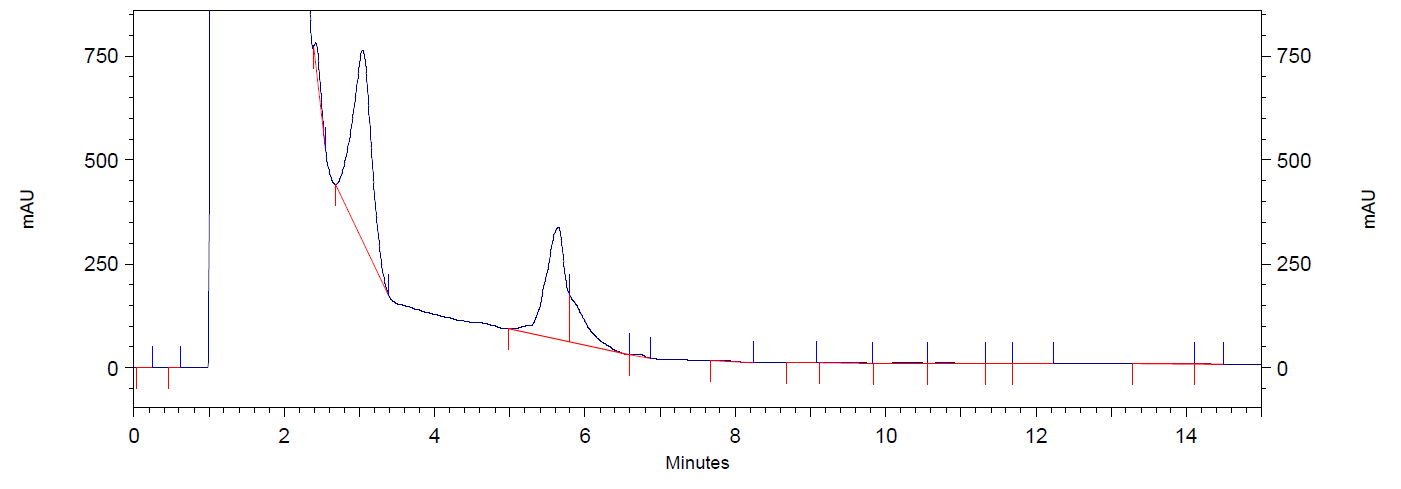


UV

Radioactivity


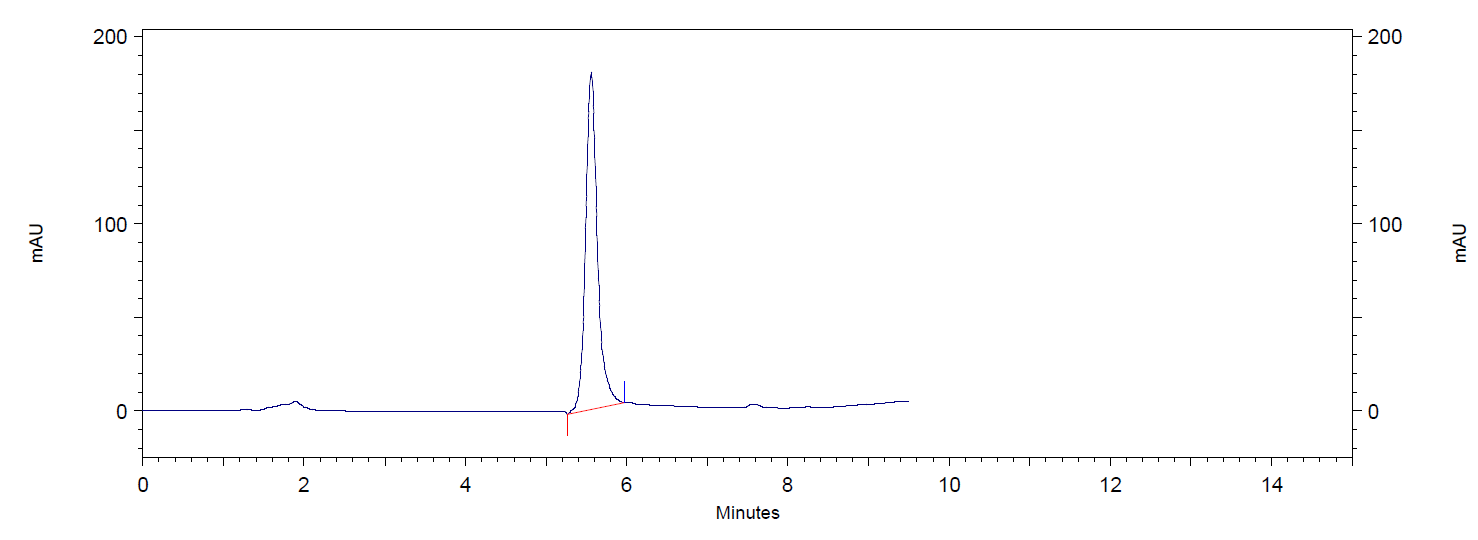


Reference UV

**4‑[^18^F]fluorobenzene**


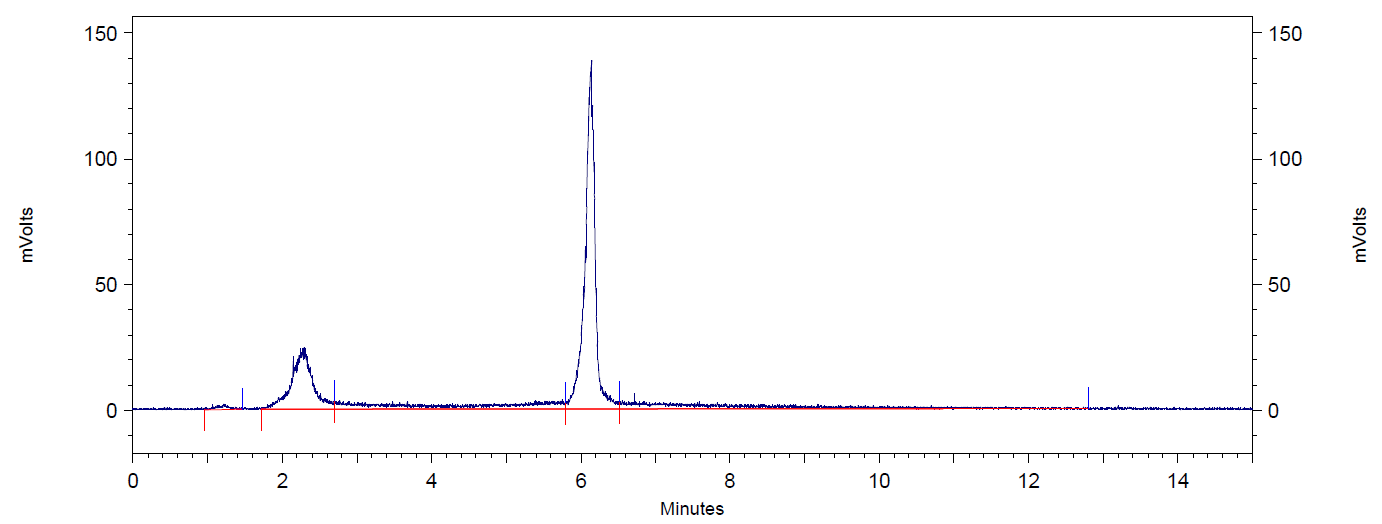

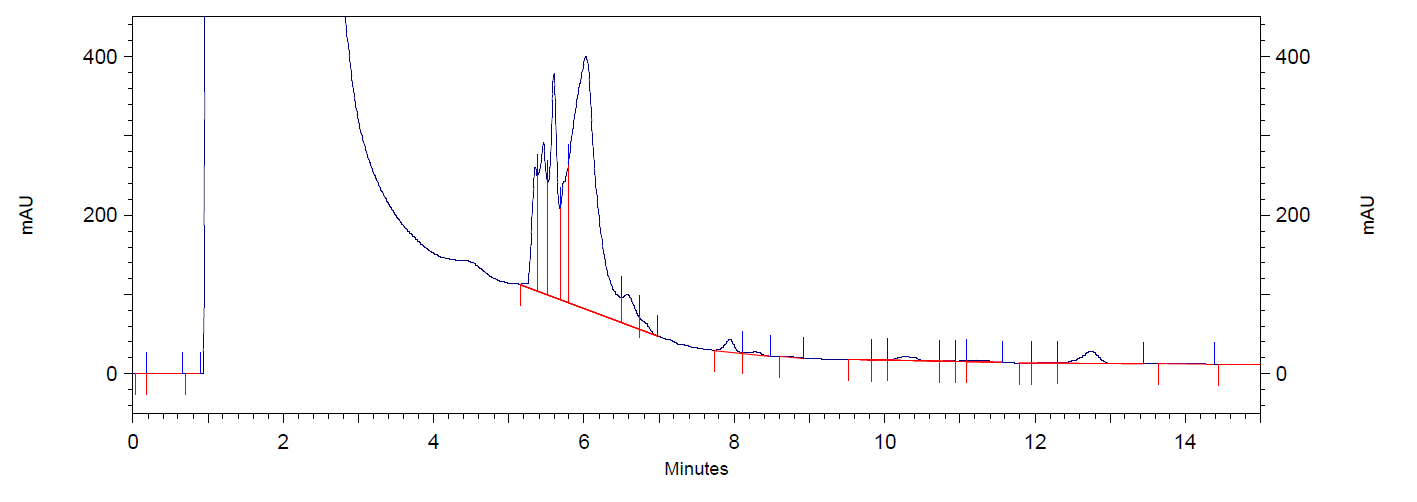


UV

Radioactivity


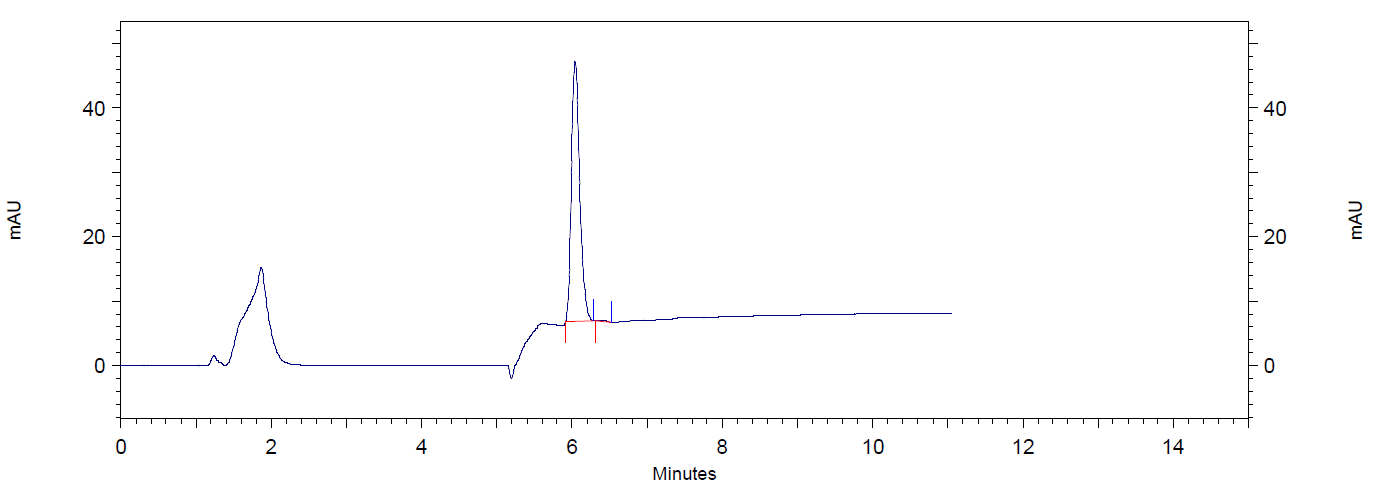


Reference UV

**4‑[^18^F]fluorobenzonitrile**


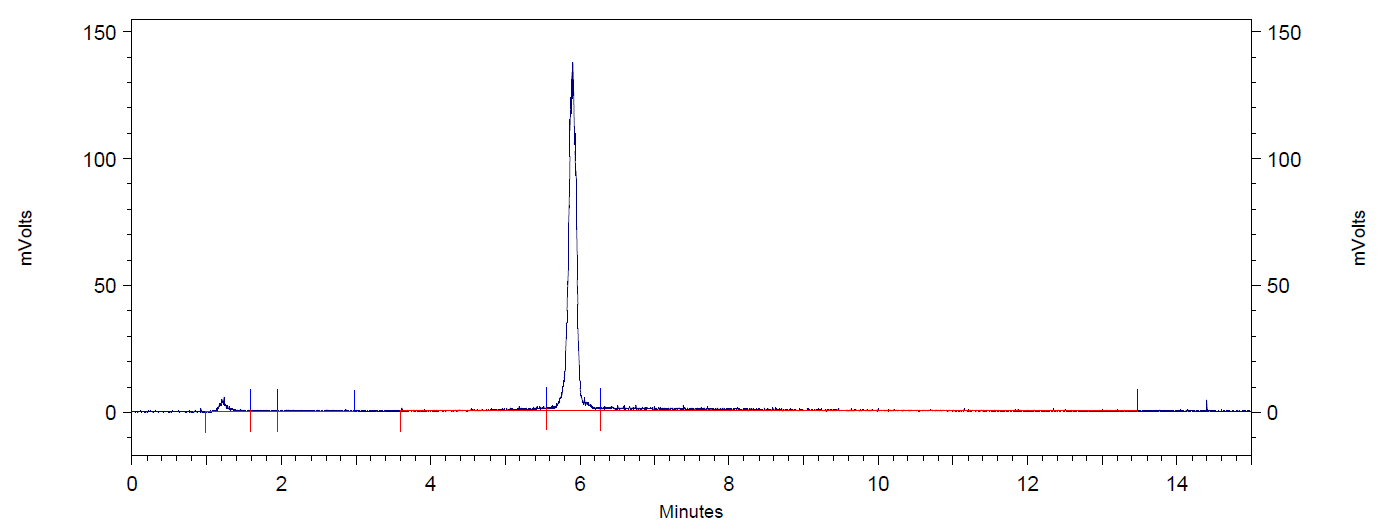

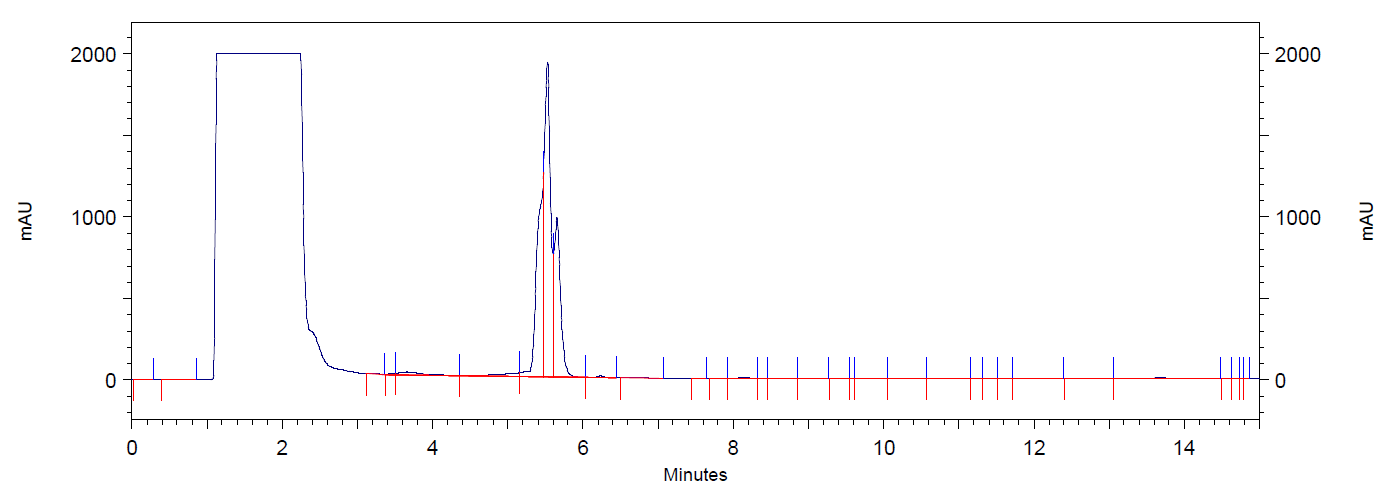


UV

Radioactivity


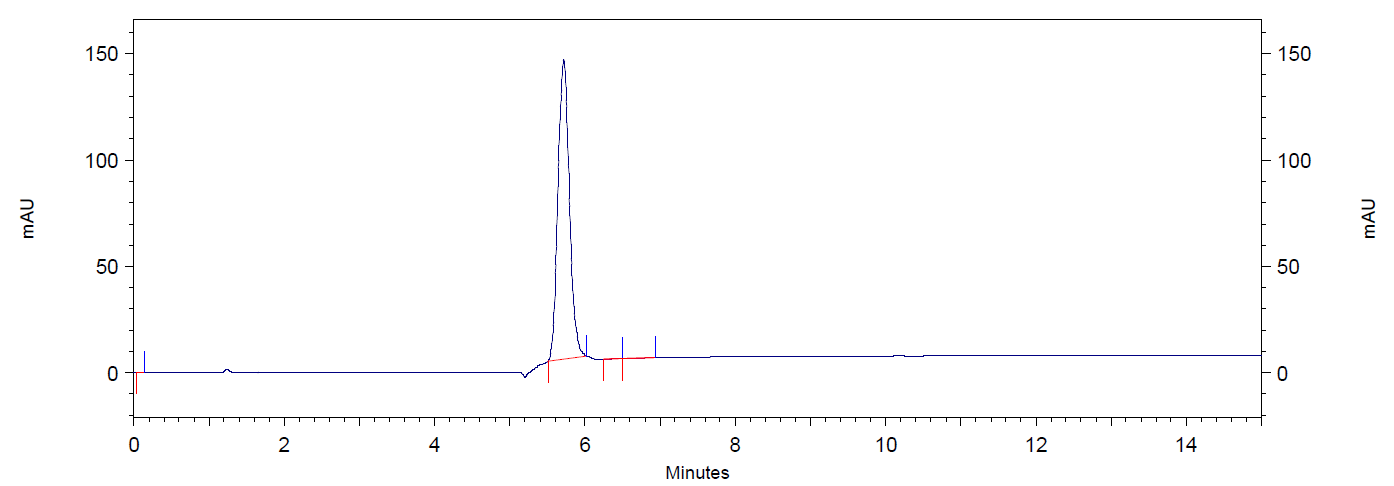


Reference UV

**3‑[^18^F]fluoropyridine**


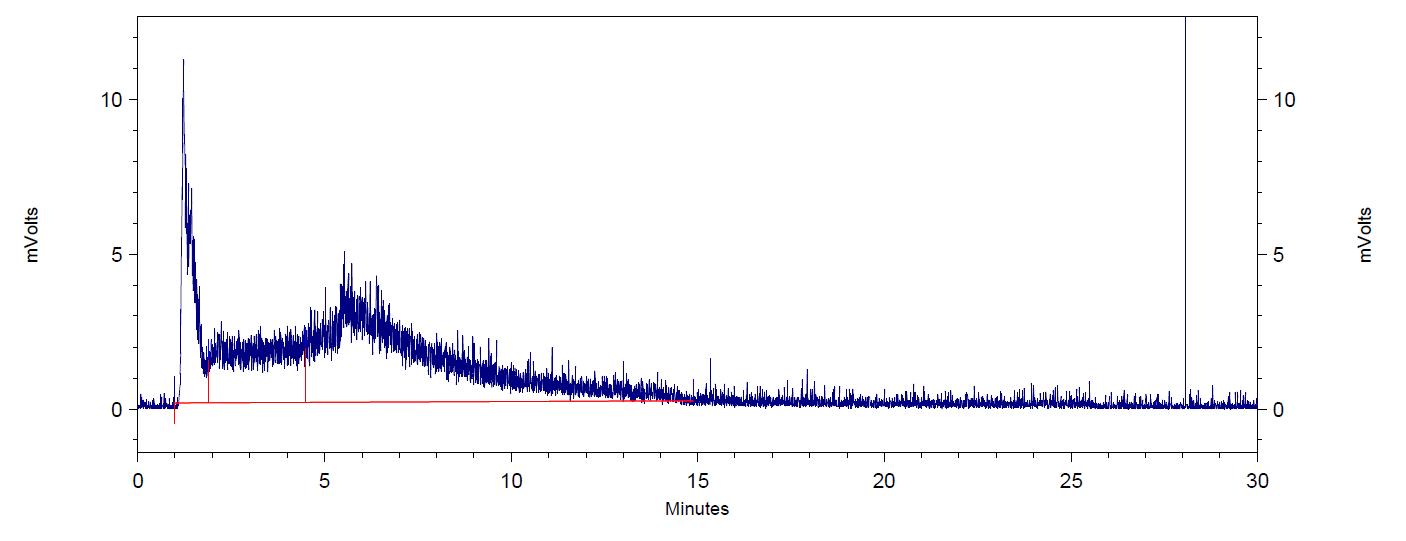

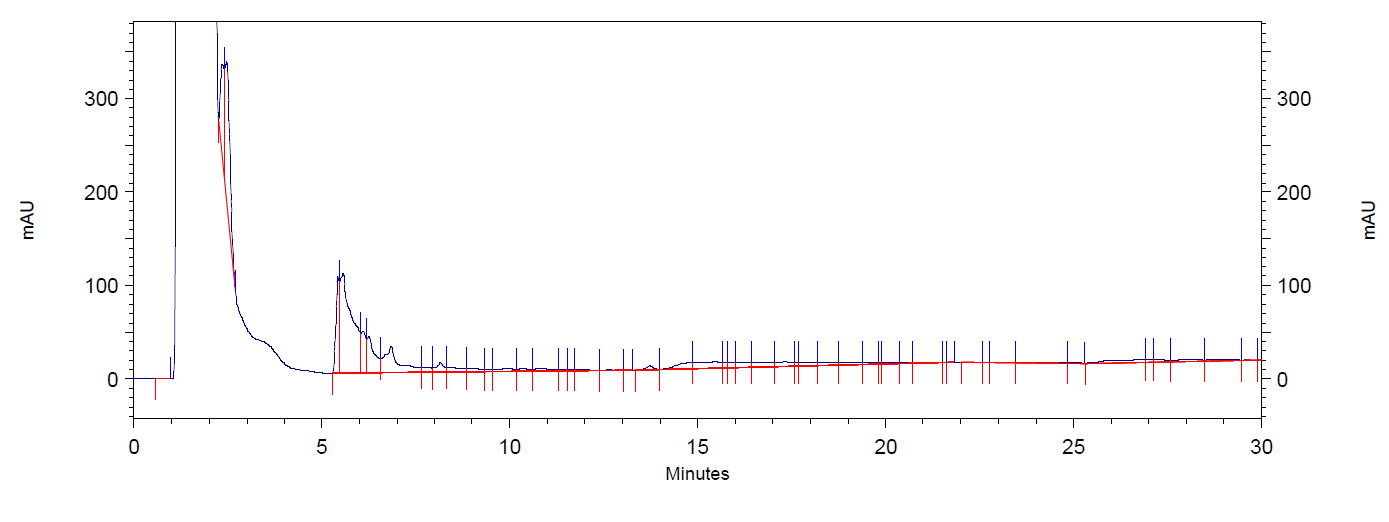


Radioactivity

UV


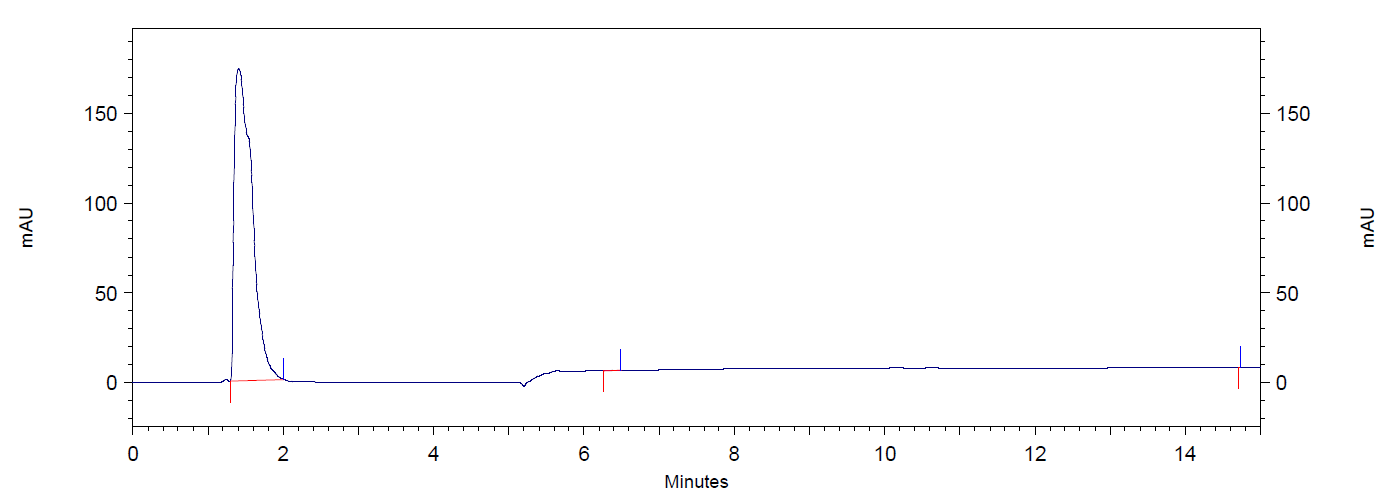


Reference UV

**2‑[^18^F]fluoronaphthalene**


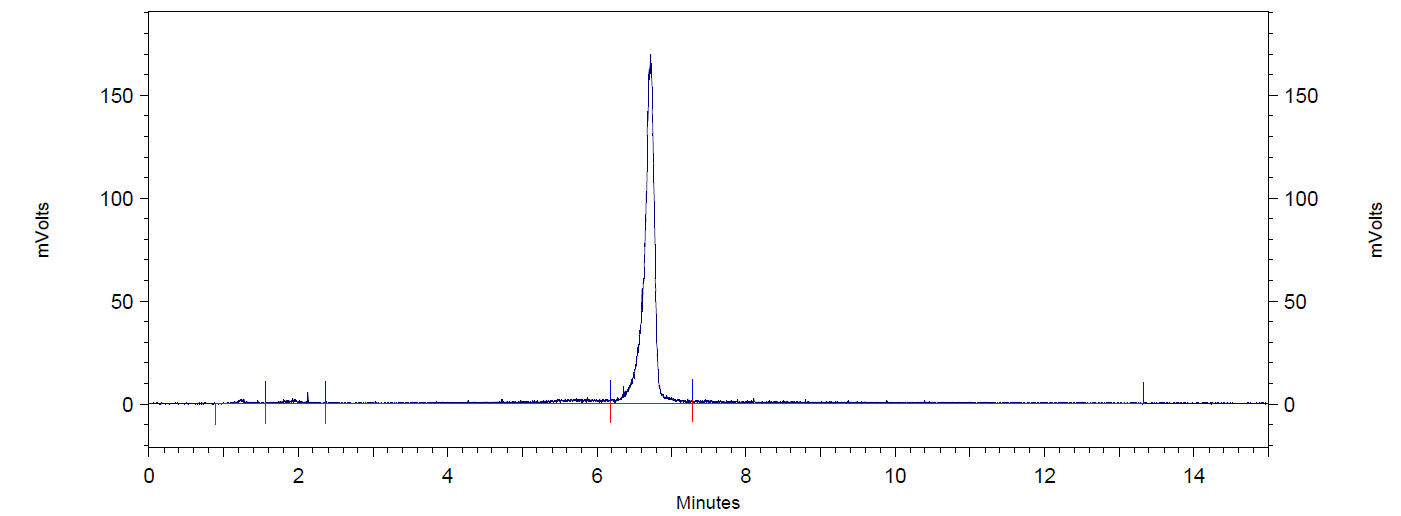

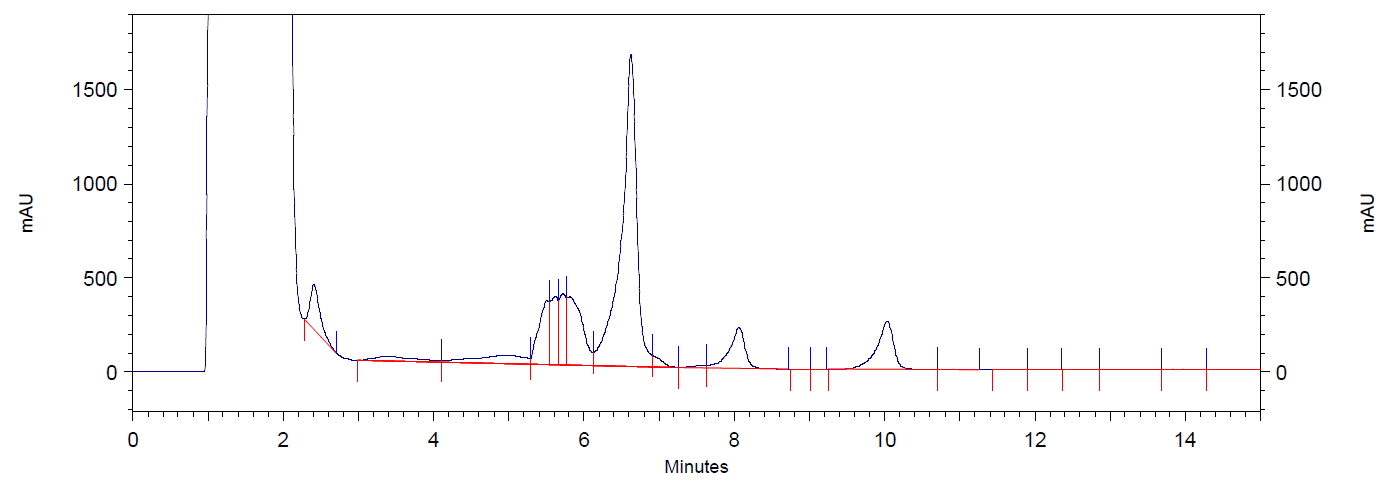


Radioactivity

UV


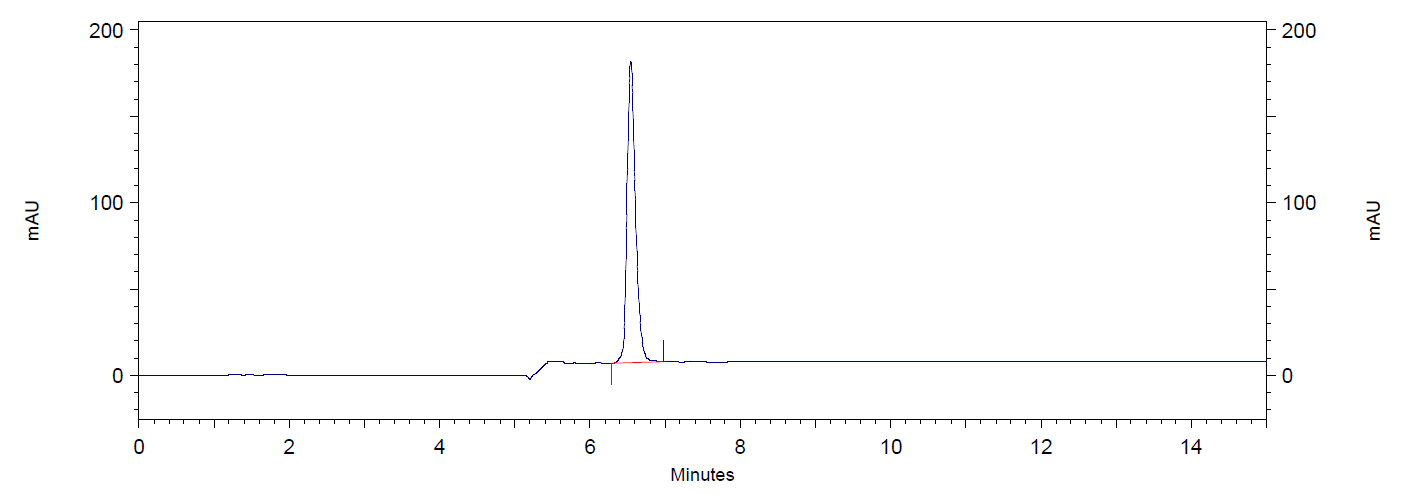


Reference UV

**4‑[^18^F]fluoroindole**


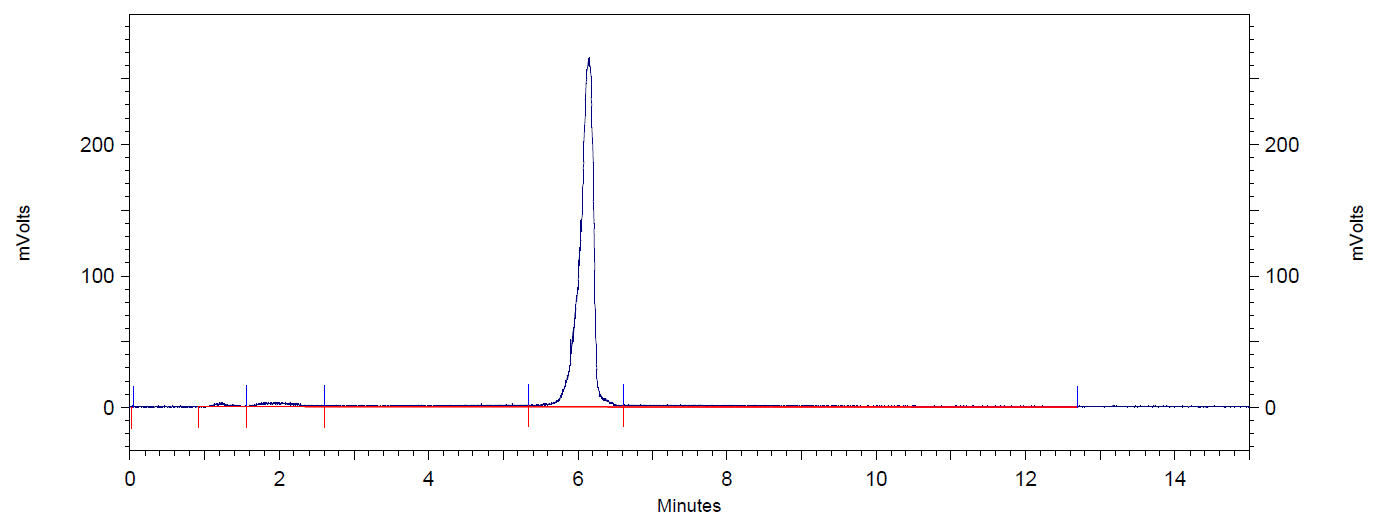

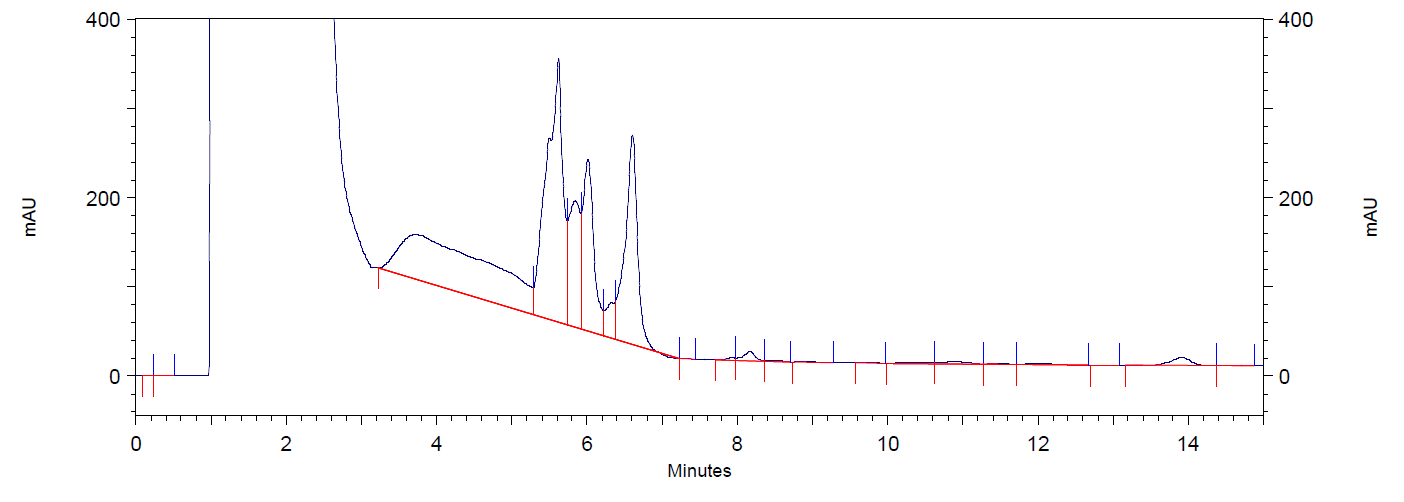


Radioactivity

UV


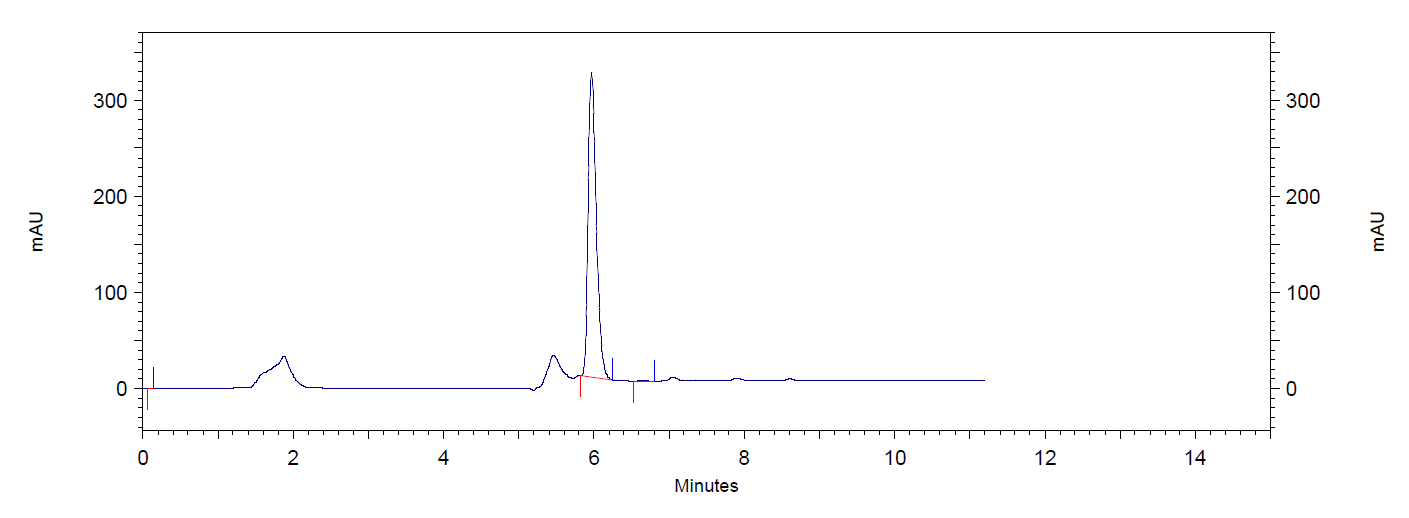


Reference UV

# Abbreviations

DMA Dimethylacetamide

HPLC High pressure liquid chromatography

RCP Radiochemical purity

RCY Radiochemical yield

SPE Solid phase extraction

THF Tetrahydrofuran

QMA Quaternary methyl ammonium

# References

1. Ametamey SM, Halldin C, Swahn C, Hall H, Schubiger PA, Farde L. Synthesis of nor-β-CIT, β-CIT and trimethylstannyl-β-CT. Nucl Med Bio. 1995;22:959-64.

2. Kirjavainen AK, Forsback S, López-Picón FR, Marjamäki P, Takkinen J, Haaparanta-Solin M, et al. ^18^F-labeled norepinephrine transporter tracer [^18^F]NS12137: radiosynthesis and preclinical evaluation. Nucl Med Biol. 2018;56:39-46.

3. Lahdenpohja S, Keller T, Rajander J, Kirjavainen AK. Radiosynthesis of the norepinephrine transporter tracer [^18^F]NS12137 via copper-mediated ^18^F-labelling. J Label Compd Radiopharm. 2019:1-6.

4. Savisto N, Viljanen T, Kokkomäki E, Bergman J, Solin O. Automated production of [^18^F]FTHA according GMP. J Label Compd Radiopharm. 2017;61:84-93.
